# Supplementary material for: Genomic regions and candidate genes associated with seed nitrogen, phosphorus, and sulfur accumulation identified in the soybean ‘Forrest’ by ‘Williams 82’ RIL population
Source: PLoS One. 2025 Sep 3;20(9):e0331214. doi: 10.1371/journal.pone.0331214 (PMC12407463; doi:10.1371/journal.pone.0331214)
Supplement: S1 File — (PDF) [file pone.0331214.s001.pdf]

Supporting information

S1 File. Genetic background of the parent used in crosses and mapping; relevant information was collected from Soybase using the below link below.. This is S1 file legend.

Link: https://legacy.soybase.org/uniformtrial/index.php?page=lines&filter=Forrest

Record and background for cultivar Forrest and Williams 82.

Record for Cultivar Forrest

Cultivar	Synonyms	Maternal Parent X Paternal Parent	Comment	Google Search (New Window)
Forrest	PI 548655<br>D68-128	Dyer **X** Bragg	PVP 7300058	Scour Google For This Line

https://legacy.soybase.org/uniformtrial/index.php?page=lines&filter=Dyer

Record for Cultivar Dyer

Cultivar	Synonyms	Maternal Parent X Paternal Parent	Comment	Google Search (New Window)
Dyer	PI 548976<br>D63-7320	Hill **X** (Lee (2) x Peking)		Scour Google For This Line

Records Containing Dyer

Cultivar	Synonyms	Maternal Parent X Paternal Parent	Comment
Coker 317		(N64-2451 x Dyer) **X** Pickett 71	
Coker 76-853	Co76-853	(N64-2451 x Dyer) **X** Pickett 71	

D66-12392		D63-6100 **X** Dyer	
D67-10507		Dyer **X** Bragg	SCN race 3 resistant selection
D68-127		Dyer **X** Bragg	
D68-128	PI 548655<br>Forrest	Dyer **X** Bragg	PVP 7300058
D68-18		Dyer **X** Bragg	
D68-180		Dyer **X** Bragg	Resistant to CN race 3
D68-201		Dyer **X** Bragg	
D68-216		Dyer **X** Bragg	later maturing, CN race 3 resistant selection
D68-78		Dyer **X** Bragg	
FFR 595	PI 561576	[(Custer x Dyer) x PI088788] **X** Bedford	FFR Cooperative, PVP 9200185
Forrest	PI 548655<br>D68-128	Dyer **X** Bragg	PVP 7300058
HSC 591	PI 561578	[(Custer x Dyer) x PI088788] **X** Bedford	Advanta USA, Inc., PVP 9200187
L78L-449		[((Hill x Dyer) x (Clark x Harosoy)] **X** Essex	
PI 556819	Terra-Vig 808	[(N64-2451 x Dyer) x Pickett 71] **X** Mack	Terral-Norris Seed Company, Inc., PVP 8600033
PI 561576	FFR 595	[(Custer x Dyer) x PI088788] **X** Bedford	FFR Cooperative, PVP 9200185
PI 561578	HSC 591	[(Custer x Dyer) x PI088788] **X** Bedford	Advanta USA, Inc., PVP 9200187
Pioneer P9571		(Dyer x Forrest) **X** J74-45	

Terra-Vig 808	PI 556819	[(N64-2451 x Dyer) x Pickett 71] **X** Mack	Terral-Norris Seed Company, Inc., PVP 8600033

……………………………

https://legacy.soybase.org/uniformtrial/index.php?page=lines&filter=Bragg

Record for Cultivar Bragg

**Cultivar**	**Synonyms**	**Maternal Parent X Paternal Parent**	**Comment**	**Google Search (New Window)**
Bragg	F58-3786<br>PI 548660	Jackson **X** D49-2491		Scour Google For This Line

Records Containing Bragg

**Cultivar**	**Synonyms**	**Maternal Parent X Paternal Parent**	**Comment**
4-74-6-3		Forrest (2) **X** [Govan x (Bragg x PI 229358)]	Insect resistant selection
AP 70	PI 556553	Bragg **X** Davis	Novartis Seeds, Inc., PVP 7800081
Asgrow A7372	PI 556686	N63-1206 **X** Bragg	Monsanto Technology, LLC, PVP 8200087
Braxton	F71-1180<br>PI 548659	F59-1505 **X** (Bragg (3) x D69-7965)	PVP 8000075
Brooks	PI 556545	Bragg **X** Hood	Gold Kist, Inc., PVP 7700025
Coker 338	Co68-38<br>PI 556515<br>Co338	Hampton 266 **X** Bragg	Coker's Pedigreed Seed Co.

Coker 488	Co73-410<br>PI 556537<br>Co488	Hampton 266 **X** Bragg	
Coker 68-38	Co68-38	Hampton 266 **X** Bragg	
Coker 68-41	Co68-41	Hampton 266 **X** Bragg	
Coker 69-119	Co69-119	Hampton 266 **X** Bragg	
Coker 71-211	Co71-211	Hampton 266 **X** Bragg	
Coker 72-286	Co72-286	Hampton 266 **X** Bragg	
Coker 73-410	Coker 488<br>PI 556537	Hampton 266 **X** Bragg	PVP 7800035
Coker 73-473	Co73-473	Hampton 266 **X** Bragg	
Coker 80-764	Coker 485<br>Co80-764	Centennial **X** [(Hampton 266 x Bragg) x Hutton]	
D66-8556		Bragg **X** Semmes	
D66-8666	Govan<br>PI 548979	Bragg **X** Semmes	
D67-10507		Dyer **X** Bragg	SCN race 3 resistant selection
D68-127		Dyer **X** Bragg	
D68-128	PI 548655<br>Forrest	Dyer **X** Bragg	PVP 7300058
D68-18		Dyer **X** Bragg	

D68-180		Dyer **X** Bragg	Resistant to CN race 3
D68-201		Dyer **X** Bragg	
D68-216		Dyer **X** Bragg	later maturing, CN race 3 resistant selection
D68-78		Dyer **X** Bragg	
D69-263		Bragg (2) **X** D60-7965	
D69-442		Bragg (3) **X** D60-7965	
D71-1180		F59-1505 **X** (Bragg (3) x D60-7965)	
D73-10232		D66-8666 **X** (Bragg x PI 229358)	
D75-10169		Govan **X** (Bragg x PI 229358)	
D75-10172		Govan **X** (Bragg x PI 229358)	
DPL 345	PI 556582	Bragg **X** Mack	Monsanto Technology, LLC, PVP 8000045
DPL 3818	PI 564528	Bedford **X** Bragg	Monsanto Technology, LLC, PVP 9300021
DPL 506	PI 556636	Bragg **X** Lee 68	Monsanto Technology, LLC, PVP 8100149
F65-1753		Bragg **X** D60-8107	
F67-3673		Bragg **X** D60-8107	
F67-3944		Bragg (2) **X** D60-7965	

F67-3970		Bragg (2) **X** D60-7965	
F67-4153		Bragg (2) **X** D60-7965	
F68-1004		Bragg (3) **X** D60-7965	
F68-1018		Bragg (3) **X** D60-7965	
F68-1025		Bragg (3) **X** D60-7965	
F68-1027		Bragg (3) **X** D60-7965	
F68-1033		Bragg (3) **X** D60-7965	
F68-1180		Bragg (3) **X** D60-7965	
F68-1577		Bragg (3) **X** D60-7965	
F68-2507		Bragg (3) **X** D60-7965	
F70-3215		Bragg (3) **X** D60-7965	
F70-3796		Bragg (2) **X** D61-3498	
F71-1004		Bragg (2) **X** D60-7965	
F71-1138		F59-1505 **X** (Bragg (2) x D60-7965)	
F71-1180		F59-1505 **X** (Bragg (3) x D60-7965)	
F71-1735		Bragg (3) **X** D60-7965	
F72-6460		Bragg (2) **X** F59-2496	
F72-6745		Bragg (3) **X** D60-7965	

F72-6831		Bragg (3) **X** D60-7965	
F73-6041		F59-1505 **X** (Bragg (2) x PI 96035)	
F73-7082		Bragg (3) **X** D60-7965	
F73-7377		F59-1505 **X** (Bragg (2) x PI 96035)	
F74-1349		F59-1505 **X** (Bragg (3) x D60-7965)	
F74-1468		F59-1505 **X** (Bragg (3) x PI 96035)	
F74-1493		F59-1505 **X** (Bragg (3) x PI 96035)	
F74-1497		F59-1505 **X** (Bragg (3) x PI 96035)	
F74-1518		F59-1505 **X** (Bragg (3) x PI 96035)	
FFR 695	PI 548834	[6041 x (6084 x Bragg)] **X** Centennial	FFR Cooperative, PVP 9100130
Forrest	PI 548655<br>D68-128	Dyer **X** Bragg	PVP 7300058
Ga72-663	Wright<br>PI 553042	Bragg **X** Lee	
Ga72-666		Bragg **X** Lee	
Ga76-316		Bragg **X** Ransom	
GaSoy 17	GaT71-1088,PI 553046	Bragg **X** Hood	
GaT71-1088	GaSoy 17<br>PI 553046	Bragg **X** Hood	

GaT74-25		Bragg **X** F66-242	
GK-67	PI 556716	Bragg **X** PI253662	Agratech Seeds Inc., PVP 8200142
Govan	D66-8666<br>PI 548979	Bragg **X** Semmes	
Gregg	La74-4656<br>F11<br>PI 510675	Bragg **X** Pickett 71	
La74-4656	Gregg<br>PI 510675	Bragg **X** Pickett 71	
McNair 700	PI 556648	Bragg **X** Ransom	Northrup King Company, PVP 7900093
N67-3831		Dare **X** Bragg	
PI 548834	FFR 695	[6041 x (6084 x Bragg)] **X** Centennial	FFR Cooperative, PVP 9100130
PI 556516	Terra-Vig 708	Coker Hampton 266 **X** Bragg	Terral-Norris Seed Company, Inc., PVP 7605003
PI 556545	Brooks	Bragg **X** Hood	Gold Kist, Inc., PVP 7700025
PI 556553	AP 70	Bragg **X** Davis	Novartis Seeds, Inc., PVP 7800081
PI 556571	Wilstar 790	Bragg **X** Ransom	Helena Chemical Company, PVP 7900058
PI 556582	DPL 345	Bragg **X** Mack	Monsanto Technology, LLC, PVP 8000045
PI 556636	DPL 506	Bragg **X** Lee 68	Monsanto Technology, LLC, PVP 8100149

PI 556648	McNair 700	Bragg **X** Ransom	Northrup King Company, PVP 7900093
PI 556686	Asgrow A7372	N63-1206 **X** Bragg	Monsanto Technology, LLC, PVP 8200087
PI 556716	GK-67	Bragg **X** PI253662	Agratech Seeds Inc., PVP 8200142
PI 564528	DPL 3818	Bedford **X** Bragg	Monsanto Technology, LLC, PVP 9300021
R69-1151		Davis **X** Bragg	
R70-580		Davis **X** Bragg	
R71-72		(Bragg x Davis) **X** (Dare x Davis)	
R81-266		Bragg **X** Centennial	
Sampson	PI 556817	Bragg **X** Essex	PVP 8600068
T70-4		Bragg **X** PI 200492	
Terra-Vig 708	PI 556516	Coker Hampton 266 **X** Bragg	Terral-Norris Seed Company, Inc., PVP 7605003
Ts70-4		Bragg **X** PI 200492	
Ts72-6		Bragg **X** PI 200492	
Wilstar 790	PI 556571	Bragg **X** Ransom	Helena Chemical Company, PVP 7900058
Wright	Ga72-663<br>PI 553042	Bragg **X** Lee	

…………………………………………..

Williams 82

https://legacy.soybase.org/uniformtrial/index.php?filter=Williams+82&page=lines&test=ALL

Record for Cultivar Williams 82

Cultivar	Synonyms	Maternal Parent X Paternal Parent	Comment	Google Search (New Window)
Williams 82	PI 518671<br>L24A	Williams (7) **X** Kingwa		Scour Google For This Line

Records Containing Williams 82

Cultivar	Synonyms	Maternal Parent X Paternal Parent	Comment
(HC)Gnome	Gnome 85	Gnome (6) **X** Williams 82	
17D		Williams 82 **X** PI 283327	Williams 82 was mutagenized
82266-B84-09240		Williams 82 **X** Asgrow A3127	
A Elgin BC	PI 518666<br>Elgin 87	Elgin (5) **X** Williams 82	PVP 8800086
A Hardin BC(k)		Hardin (5) **X** Williams 82	
A Harper BC	PI 518667<br>Harper 87	Harper (6) **X** Williams 82	PVP 8800087
ABSR 101BC	PI 546487<br>Archer	(BSR 101 (5) x Williams 82) **X** [BSR 101 (5) x (Harosoy x Altona)]	PVP 9100040

AHW-Pella BC	PI 509044<br>Pella 86	Pella (5) **X** Williams 82	
Amcor 89	PI 546375	Amcor **X** Williams 82	
Archer	PI 546487<br>ABSR 101BC<br>BSR 101BC	(Williams 82 x BSR 101) **X** (PRX 54-59 x BSR 101)	PVP 9100040, See PI 546487 for parentage explaination
Asgrow A2234	A2234<br>PI 556850	(Calland x Amsoy) **X** (Century (3) x Williams 82)	PVP 008700079
Asgrow A3501	PI 556573	Williams 82 **X** Asgrow A3127	Monsanto Technology, LLC, PVP 8700119
Asgrow A3511	PI 556815	Williams 82 **X** Asgrow A3127	Monsanto Technology, LLC, PVP 8600163
C1747		A80-244003 **X** Williams 82	
Century 84	PI 548529<br>HW8185	Century (5) **X** Williams 82	PVP 8500058
CX291	PI 547094	Asgrow A3127 **X** Williams 82	Monsanto Technology, LLC, PVP 9100075
CX298	PI 556888	Williams 82 **X** Asgrow A3127	Monsanto Technology, LLC, PVP 8800188
CX329	PI 556931	Williams 82 **X** Asgrow A3127	Monsanto Technology, LLC, PVP 9000005
DS5-2675		Williams 82 **X** Ankur	Rust (50% exotic pedigree)

DS5-67		Williams 82 **X** Ankur	Rust resistance
Elgin 87	PI 518666<br>A Elgin BC	Elgin (5) **X** Williams 82	PVP 8800086
Elgin BC	Elgin 87<br>A Elgin BC	Elgin (5) **X** Williams 82	
FFR 253	PI 559397	Williams 82 **X** Pella	FFR Cooperative, PVP 9200055
FFR 373	PI 559400	Williams 82 **X** Pella	FFR Cooperative, PVP 9200058
Flyer	PI 534646<br>HM8469<br>A3127BC3F2-10'	Asgrow A3127 (4) **X** Williams 82	PVP 8700125; Literature says Asgrow A3127 x L24. L24 is closely related to L24A which was released as Williams 82. PVP says Asgrow A3127 x Williams 82.
Gnome 85	PI 543857<br>Gnome Rps1-k<br>HC Gnome Rps1-k	Gnome (6) **X** Williams 82	PVP 8700097
Gnome Rps1-k	PI 543857<br>Gnome 85<br>HC Gnome Rps1-k	Gnome (6) **X** Williams 82	PVP 8700097
GP-164	LN89-5717	Williams 82 (2) **X** PI 89772	F3, Resistant to SCN Races 2, 3, 5 and 14.

GR8836	PI 534647	Asgrow A3127 **X** Williams 82	
GR8936	PI 534648<br>OX79104<br>HM8486	Asgrow A3127 **X** Williams 82	Ohio Agricultural Research and Development Center, Ohio State University, PVP 8700128
Hardin 91		Hardin (5) **X** Williams 82	
Harper 87	PI 518667<br>A Harper BC	Harper (6) **X** Williams 82	PVP 8800087
Harper BC	A Harper BC	Harper (6) **X** Williams 82	
Hayes	PI 542709<br>HM8482	Amcor **X** Williams 82	PVP 9000045
HC Amcor	Amcor 89<br>PI 546375	Amcor (6) **X** Williams 82	
HC Elf BC		Elf (6) **X** Williams 82	
HC Elf-EB		Elf (6) **X** Williams 82	
HC Gnome Rps1-k	PI 543857<br>Gnome 85<br>Gnome Rps1-k	Gnome (6) **X** Williams 82	PVP 8700097
HC Hobbit BC	PI 546373<br>Hobbit 87	Hobbit (6) **X** Williams 82	PVP 9000013
HC Sprite BC	PI 546374<br>Sprite 87	Sprite (7) **X** Williams 82	PVP 9000014

HC83-2408		Sprite **X** Williams 82	
HC83-2546		Hobbit **X** Williams 82	
HC83-3834		HC74-3400 **X** Williams 82	
HC84-4850		Sprite **X** Williams 82	
HC84-4851		Sprite **X** Williams 82	
HC84-4874		Hobbit **X** Williams 82	
HC85-2206		Elf **X** Williams 82	Parentage conflict. NUST 2012
HM8470		Asgrow A3127 (4) **X** Williams 82	
HM8471	PI 534645 Resnik	Asgrow A3127 (4) **X** Williams 82	PVP 8700126
HM8473	GR8836	Asgrow A3127 (4) **X** Williams 82	
HM8482	PI 542709 Hayes	Amcor **X** Williams 82	PVP 9000045
HM8486	GR8936 PI 534648	Asgrow A3127 **X** Williams 82	
HM8776		A80-147003 **X** (Asgrow A3127 (4) x Williams 82)	
Hobbit 87	PI 546373 HC Hobbit BC	Hobbit (6) **X** Williams 82	PVP 9000013
Hodgson 87		Hobbit (6) **X** Williams 82	

HS84-6276		Harper (3) **X** Williams 82	
HS87-4087		A81-156027 **X** (Asgrow A3127 (4) x Williams 82)	
HW8185	PI 548529<br>Century 84	Century (5) **X** Williams 82	PVP 8500058
Kunitz	PI 542044<br>L81-4590	Williams 82 (6) **X** PI 157440	
L81-4590	PI 542044<br>Kunitz	Williams 82 (6) **X** PI 157440	PVP 9000169
L82C-1246	PI 542043<br>Linford	Williams 82 **X** Fayette	CV-270
L84-6189		Williams 82 **X** L78-4245	
L85-2378	PI 547875	Williams 82 (6) **X** PI 200492	
LG01-7728		Williams 82 **X** (Williams x PI 479767)	
LG01-7812		Williams 82 **X** (Williams X PI 483461)	
LG01-7884		Williams 82 **X** (Williams x PI 549046)	
LG07-4722		PI 507807 **X** Williams 82	
LG08-3465		(Williams 82 x IA3023) **X** PI 479767	
Linford	PI 542043<br>L82C-1246	Williams 82 **X** Fayette	CV-270
LN82-3254		Williams 82 **X** Hardin	

LN82-4433		Williams 82 **X** Century	
LN89-5717	GP-164<br>PI 574542	Williams 82 (2) **X** PI 89772	F3, Resistant to SCN Races 2, 3, 5 and 14.
Pella 86	PI 509044<br>AHW-Pella BC	Pella (5) **X** Williams 82	
PI 534648	GR8936	Asgrow A3127 **X** Williams 82	Ohio Agricultural Research and Development Center, Ohio State University, PVP 8700128
PI 547094	CX291	Asgrow A3127 **X** Williams 82	Monsanto Technology, LLC, PVP 9100075
PI 547875	L85-2378	Williams 82 (6) **X** PI 200492	
PI 547878	L86-1752	Williams 82 (6) **X** PI 230970	
PI 547879	L87-0482	Williams 82 (6) **X** PI 459025	
PI 556573	Asgrow A3501	Williams 82 **X** Asgrow A3127	Monsanto Technology, LLC, PVP 8700119
PI 556815	Asgrow A3511	Williams 82 **X** Asgrow A3127	Monsanto Technology, LLC, PVP 8600163
PI 556888	CX298	Williams 82 **X** Asgrow A3127	Monsanto Technology, LLC, PVP 8800188
PI 556931	CX329	Williams 82 **X** Asgrow A3127	Monsanto Technology, LLC, PVP 9000005

PI 559397	FFR 253	Williams 82 **X** Pella	FFR Cooperative, PVP 9200055
PI 559400	FFR 373	Williams 82 **X** Pella	FFR Cooperative, PVP 9200058
PI 561583	Pioneer P9351	Williams 82 **X** Asgrow A1937	Pioneer Hi-Bred International, Inc., PVP 9200192
PI 574542	LN89-5717	Williams 82 (2) **X** PI 89772	F3, Resistant to SCN Races 2, 3, 5 and 14.
PI 578296	Syngenta S39-41	Syngenta S42-30 (2) **X** (Williams 82 x PRX58-35)	Novartis Seeds, Inc., PVP 9400140
Pioneer P9351	PI 561583	Williams 82 **X** Asgrow A1937	Pioneer Hi-Bred International, Inc., PVP 9200192
RCAT Alliance	PI 548646	Wells II **X** Williams 82	
Resnik	PI 534645<br>HM8471	Asgrow A3127 (4) **X** Williams 82	PVP 8700126
SB-01	10-73#5	Williams 82 **X** (Pana x CX1512-44)	
Sprite 87	PI 546374<br>HC Sprite BC	Sprite (7) **X** Williams 82	PVP 9000014
Syngenta S39-41	PI 578296	Syngenta S42-30 (2) **X** (Williams 82 x PRX58-35)	Novartis Seeds, Inc., PVP 9400140
TN-EXW2-051		Essex **X** Williams 82	

TN-EXW2-111		Essex **X** Williams 82	
TN-EXW2-141		Essex **X** Williams 82	
XP1928		(Hardin x Williams 82) **X** [(Tracy x Williams) x HW79149]	

Records Containing Williams (7)

https://legacy.soybase.org/uniformtrial/index.php?page=lines&filter=Williams+%287%29

**Cultivar**	**Synonyms**	**Maternal Parent X Paternal Parent**	**Comment**
L24A	PI 518671<br>Williams 82	Williams (7) **X** Kingwa	Composite of 4 Phytopthora resistant F3BC6 lines selected for similarity to Williams; CV-222
L26		Williams (7) **X** Harrel	
Williams 82	PI 518671<br>L24A	Williams (7) **X** Kingwa	
Williams BC6	PI 548585<br>Winchester	Williams (7) **X** PRX12-112	PVP 8400082
Winchester	PI 548585<br>Williams BC6	Williams (7) **X** PRX12-112	PVP 8400082

Record for Cultivar Kingwa

https://legacy.soybase.org/uniformtrial/index.php?page=lines&filter=Kingwa

Cultivar	Synonyms	Maternal Parent X Paternal Parent	Comment	Google Search (New Window)
Kingwa	PI 548359<br>Pekwa<br>T206	Selection from Peking	Rogue selection from Peking ca. 1921	Scour Google For This Line

Records Containing Kingwa

Cultivar	Synonyms	Maternal Parent X Paternal Parent	Comment
L24		Williams (??) **X** Kingwa	Williams backcross population, closely related to Williams 82
L24A	PI 518671<br>Williams 82	Williams (7) **X** Kingwa	Composite of 4 Phytopthora resistant F3BC6 lines selected for similarity to Williams; CV-222
L27		Corsoy (8) **X** Kingwa	
L78-189		Corsoy **X** Kingwa	
Williams 82	PI 518671<br>L24A	Williams (7) **X** Kingwa	
