# Supplementary figures and images for: Genomic regions and candidate genes associated with seed nitrogen, phosphorus, and sulfur accumulation identified in the soybean ‘Forrest’ by ‘Williams 82’ RIL population
Source: PLoS One. 2025 Sep 3;20(9):e0331214. doi: 10.1371/journal.pone.0331214 (PMC12407463; doi:10.1371/journal.pone.0331214)

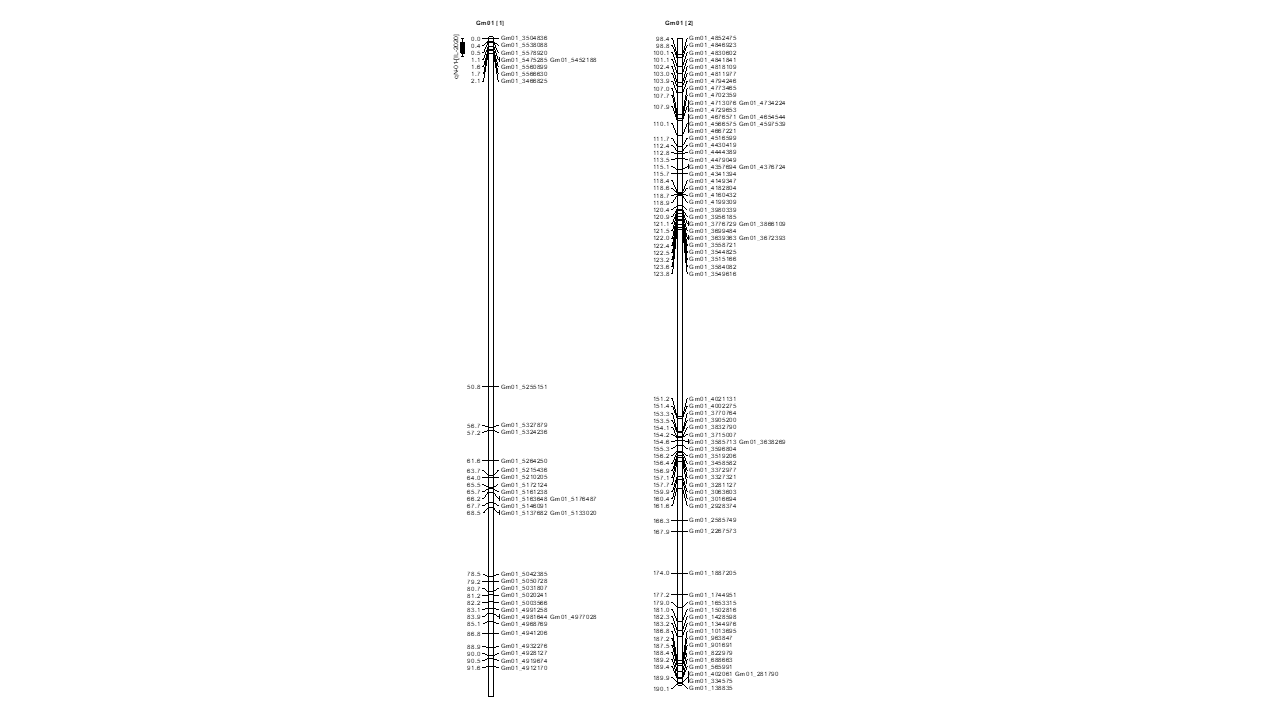

Supplement: S1 Fig — (TIF) [file pone.0331214.s004.TIF]

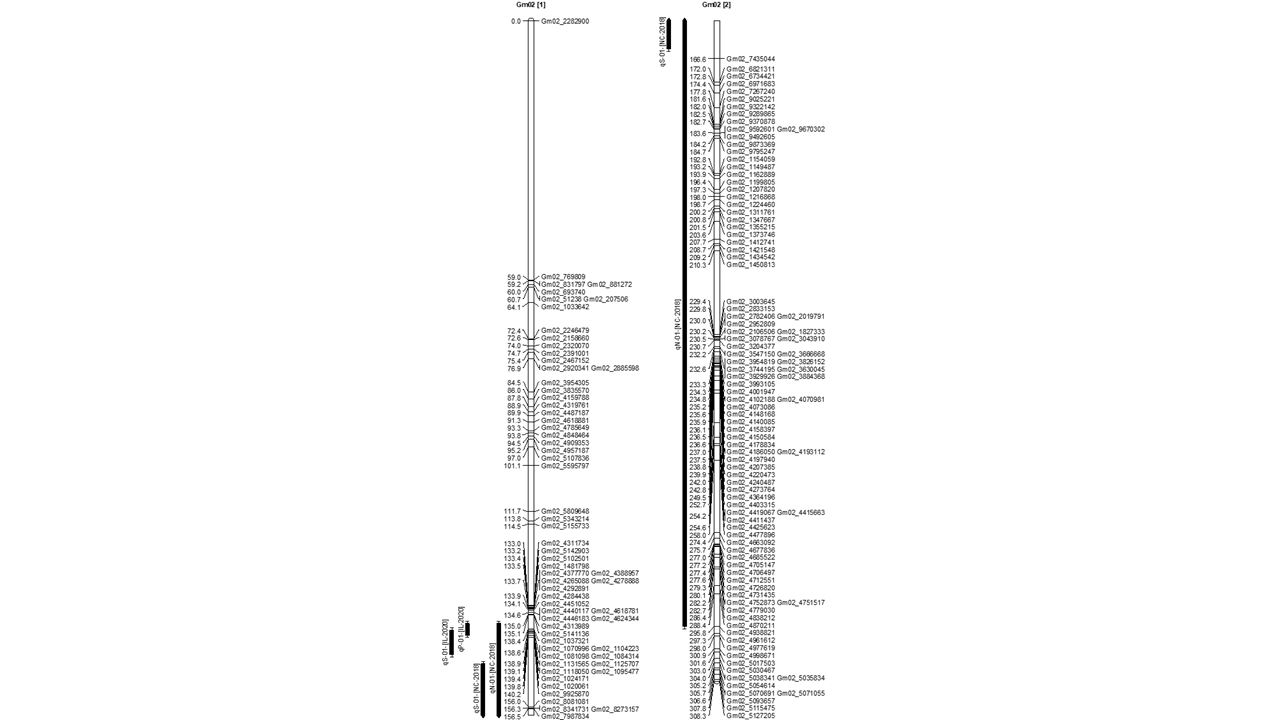

Supplement: S2 Fig — (TIF) [file pone.0331214.s005.TIF]

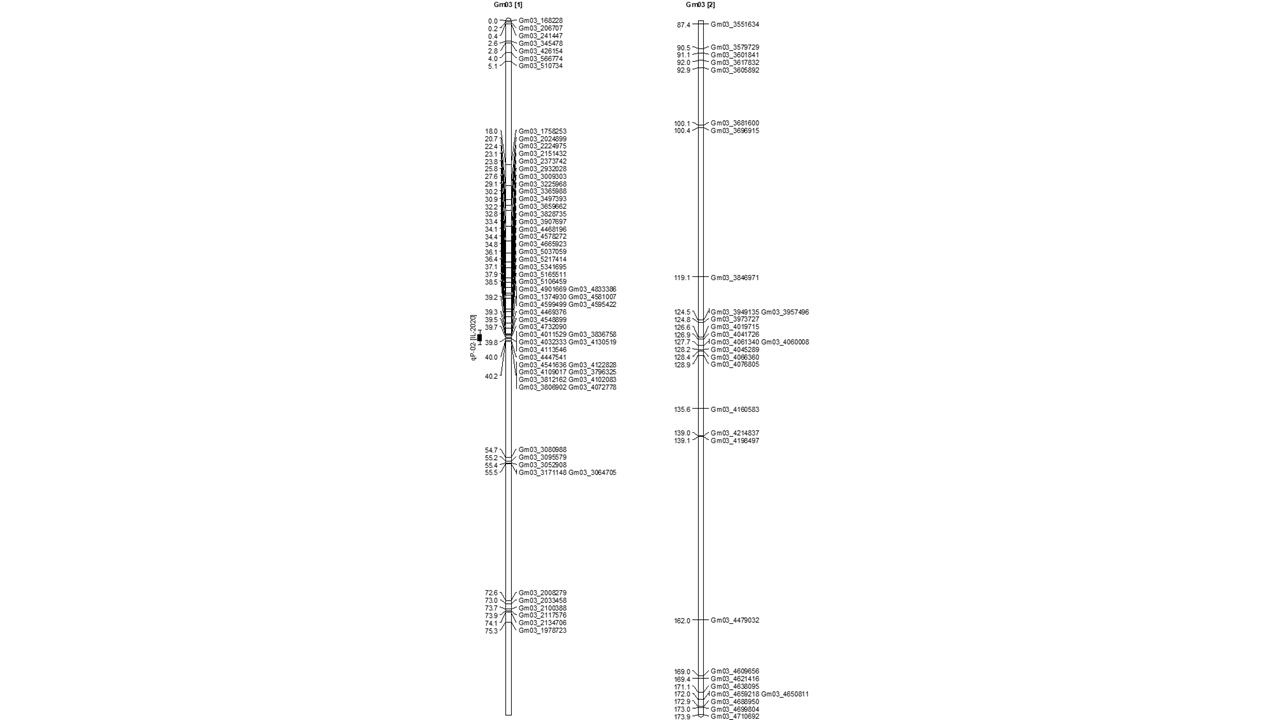

Supplement: S3 Fig — (TIF) [file pone.0331214.s006.TIF]

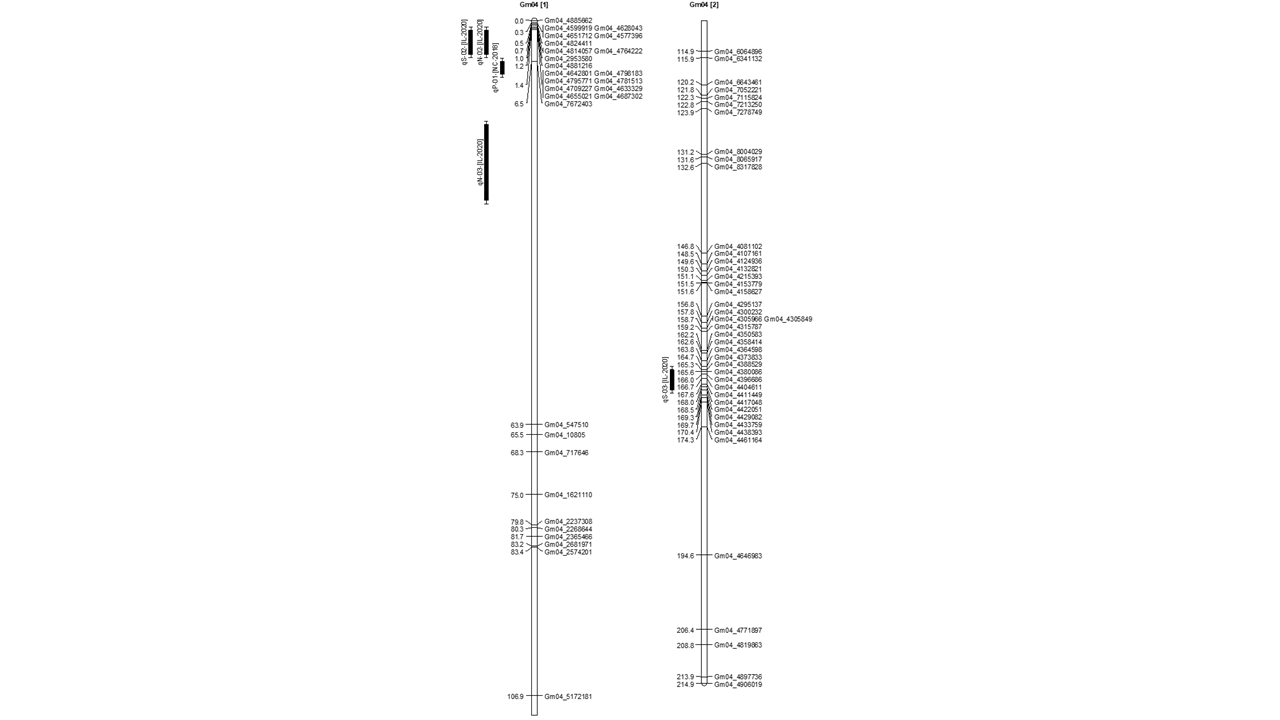

Supplement: S4 Fig — (TIF) [file pone.0331214.s007.TIF]

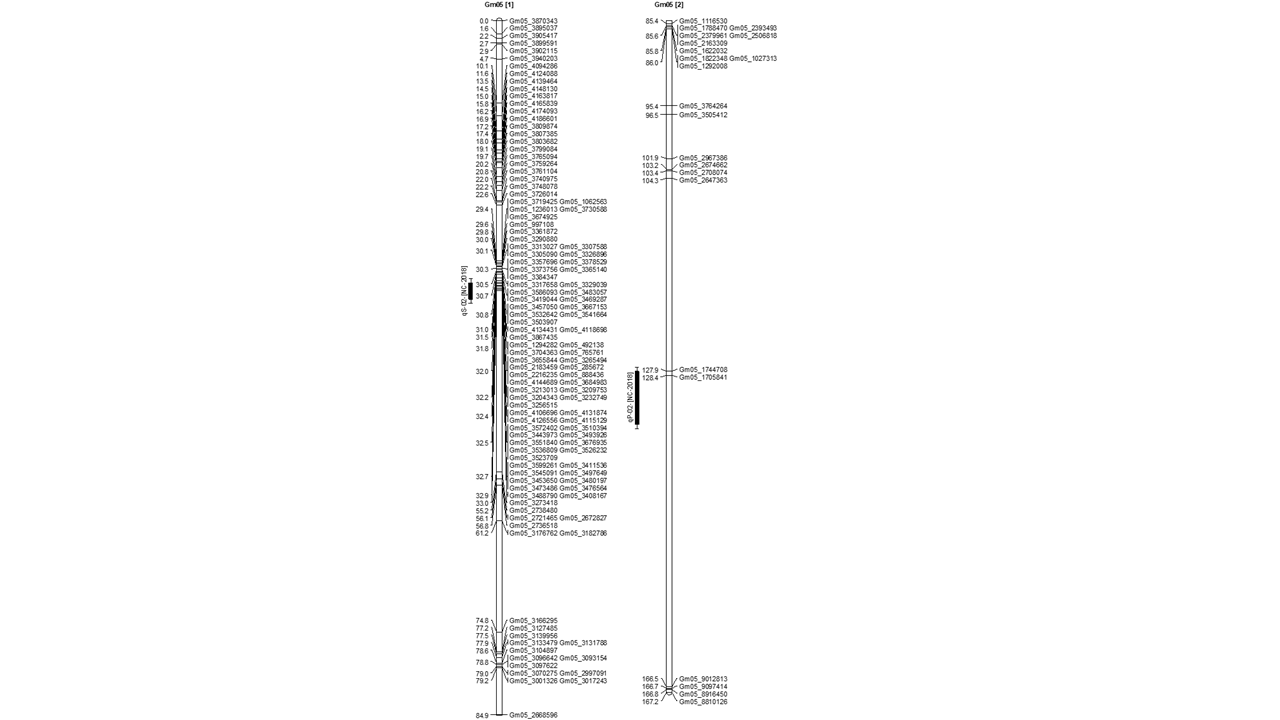

Supplement: S5 Fig — (TIF) [file pone.0331214.s008.TIF]

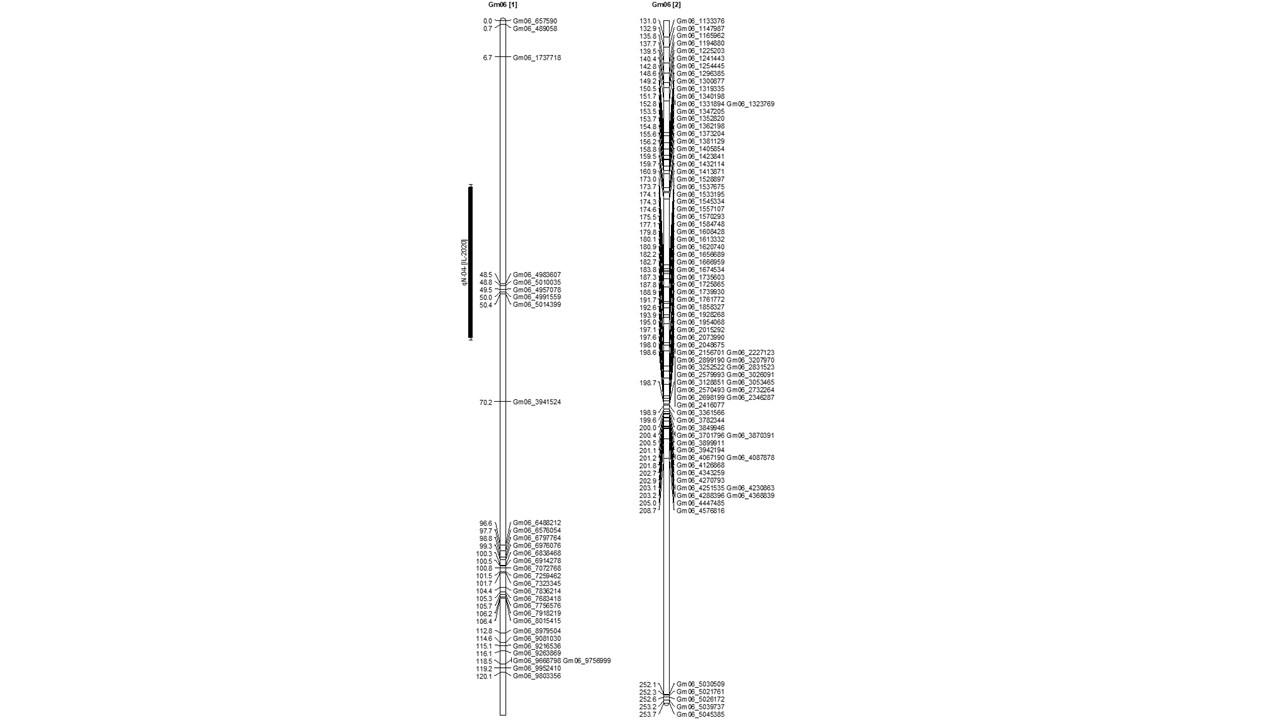

Supplement: S6 Fig — (TIF) [file pone.0331214.s009.TIF]

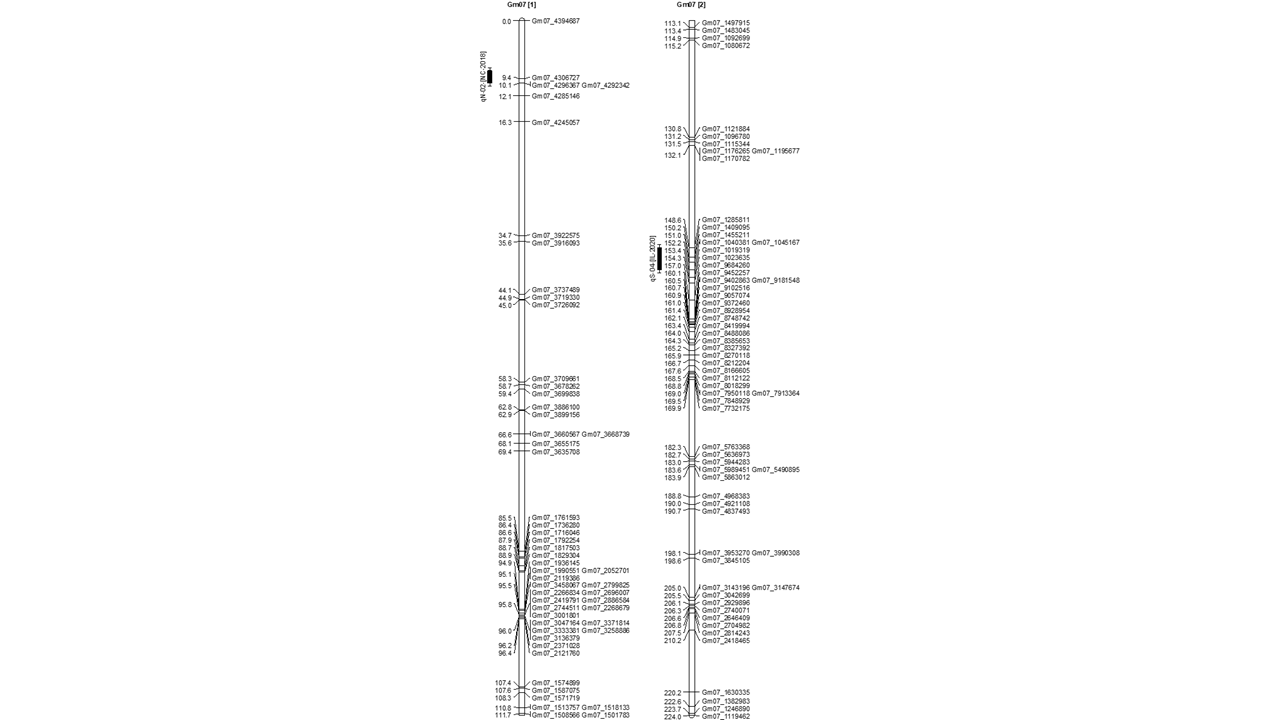

Supplement: S7 Fig — (TIF) [file pone.0331214.s010.TIF]

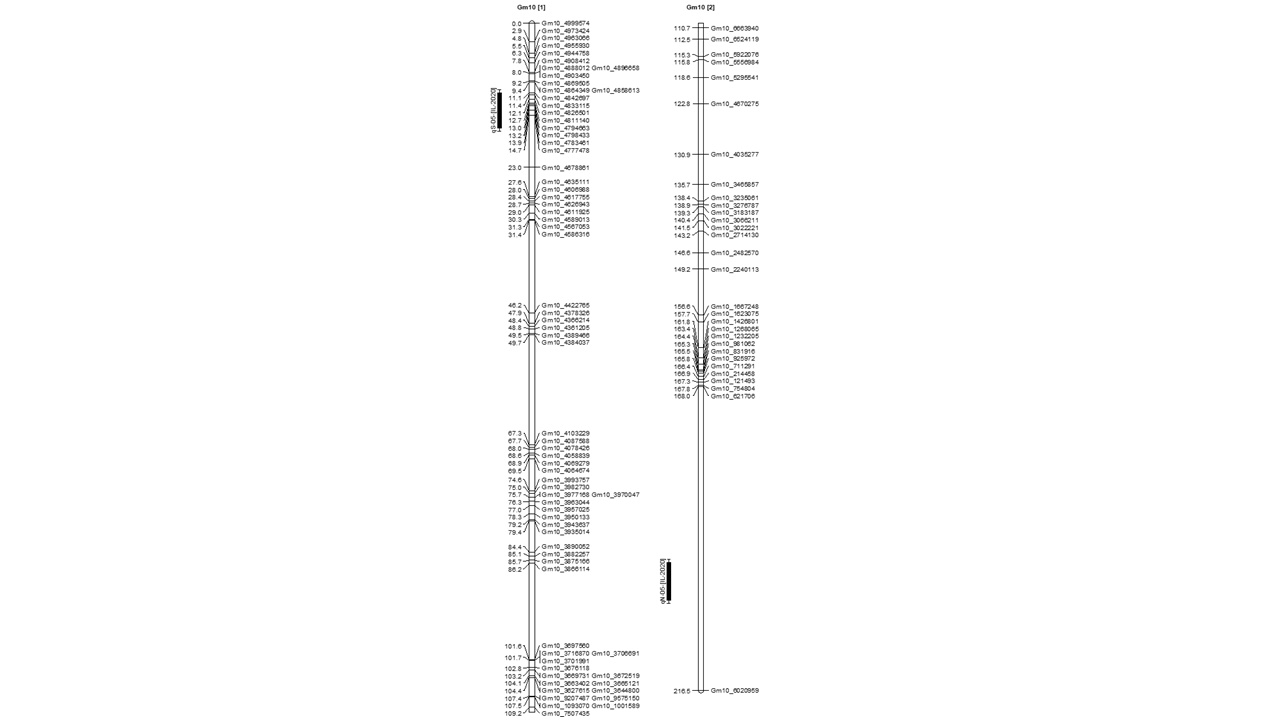

Supplement: S8 Fig — (TIF) [file pone.0331214.s011.TIF]

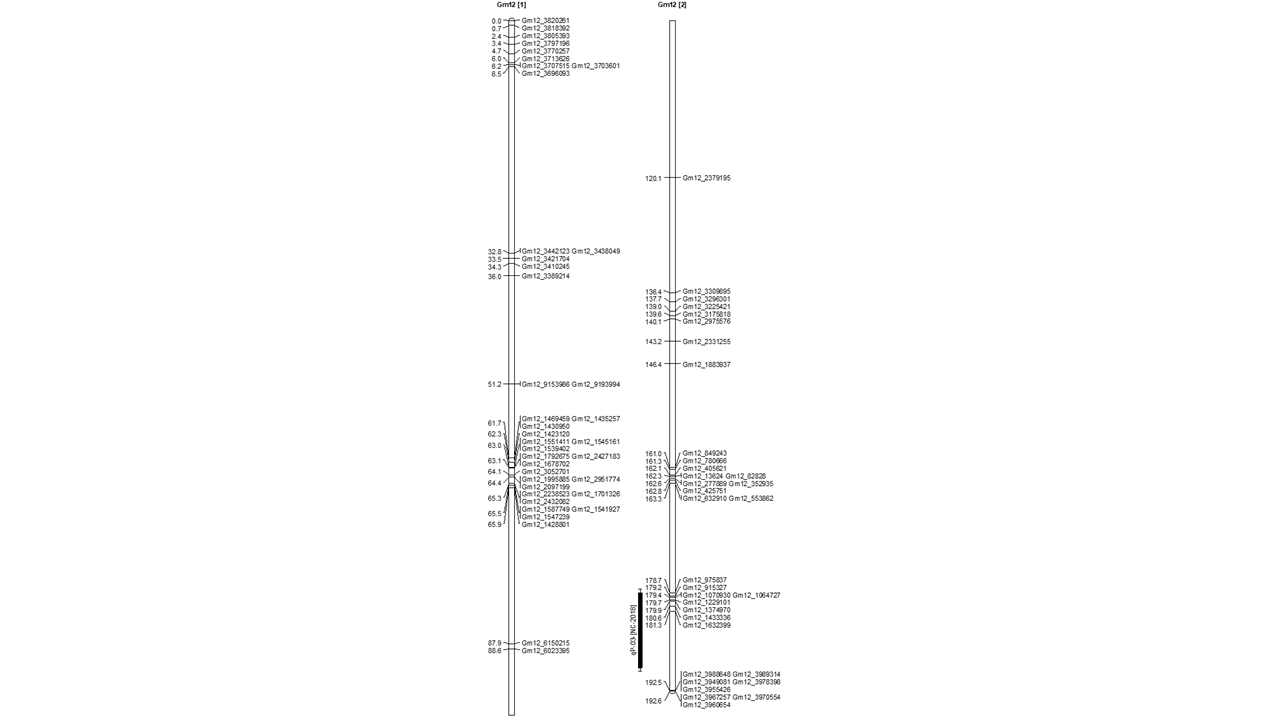

Supplement: S9 Fig — (TIF) [file pone.0331214.s012.TIF]

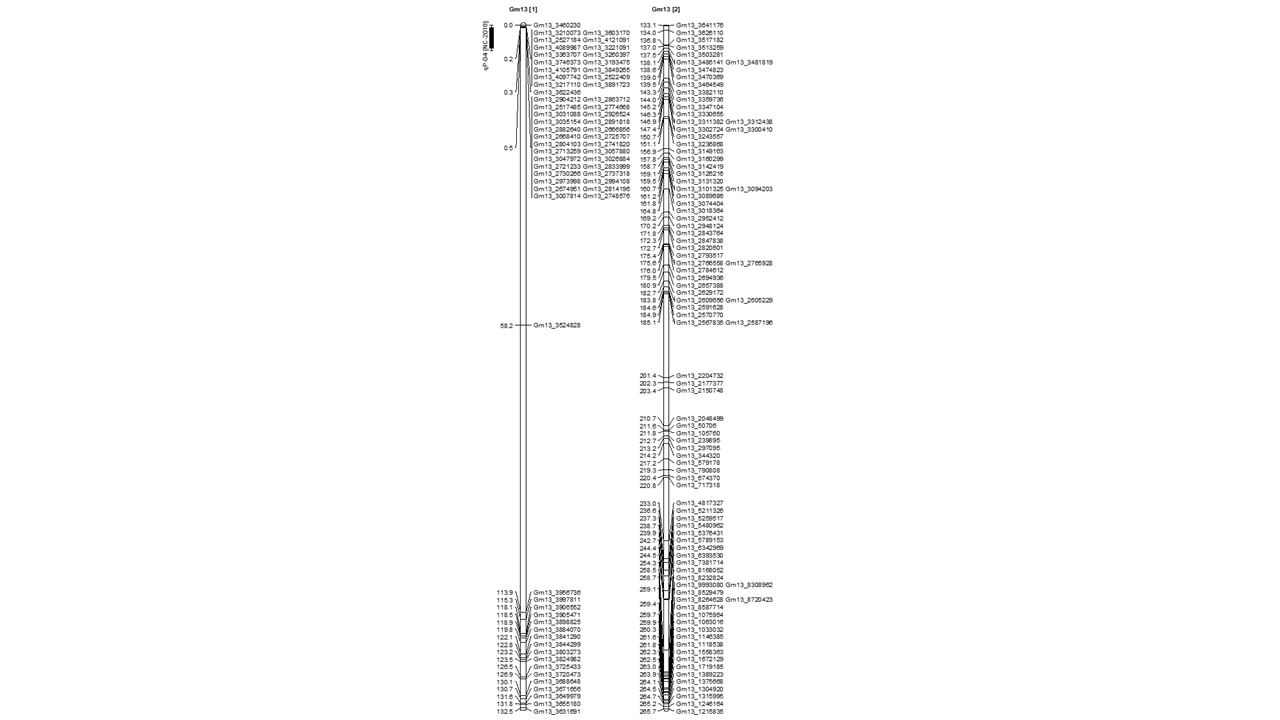

Supplement: S10 Fig — (TIF) [file pone.0331214.s013.TIF]

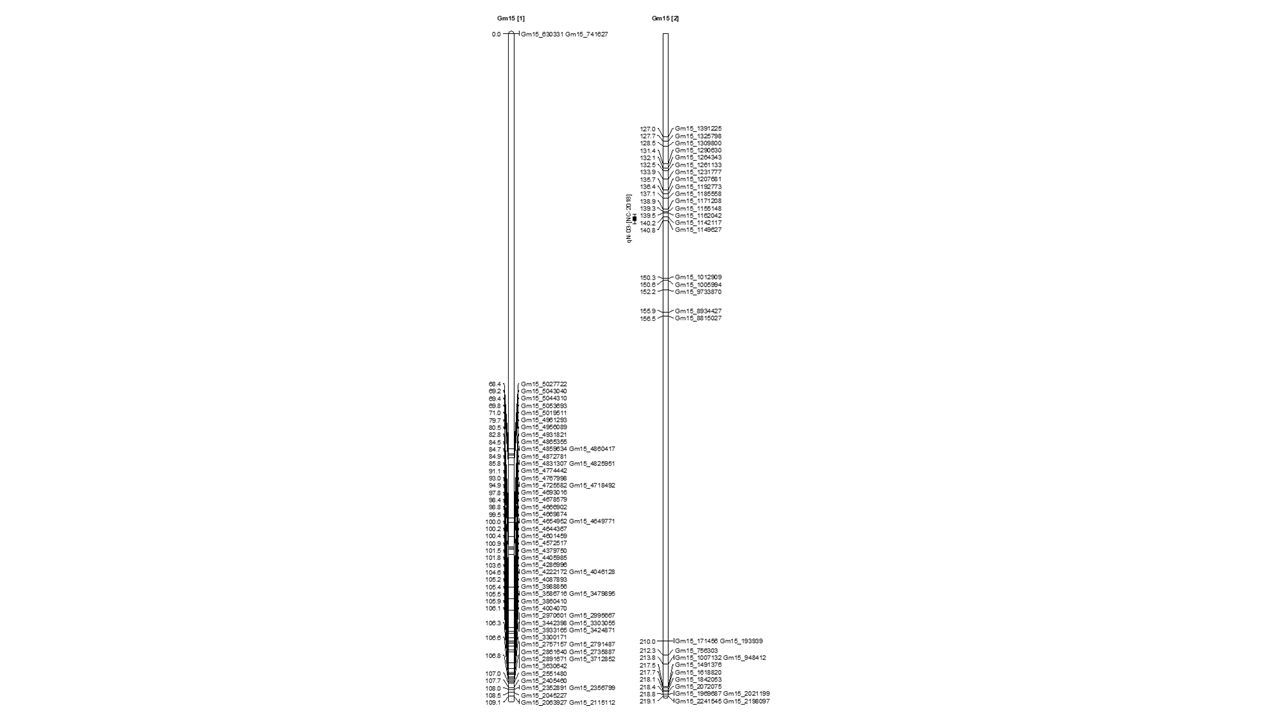

Supplement: S11 Fig — (TIF) [file pone.0331214.s014.TIF]

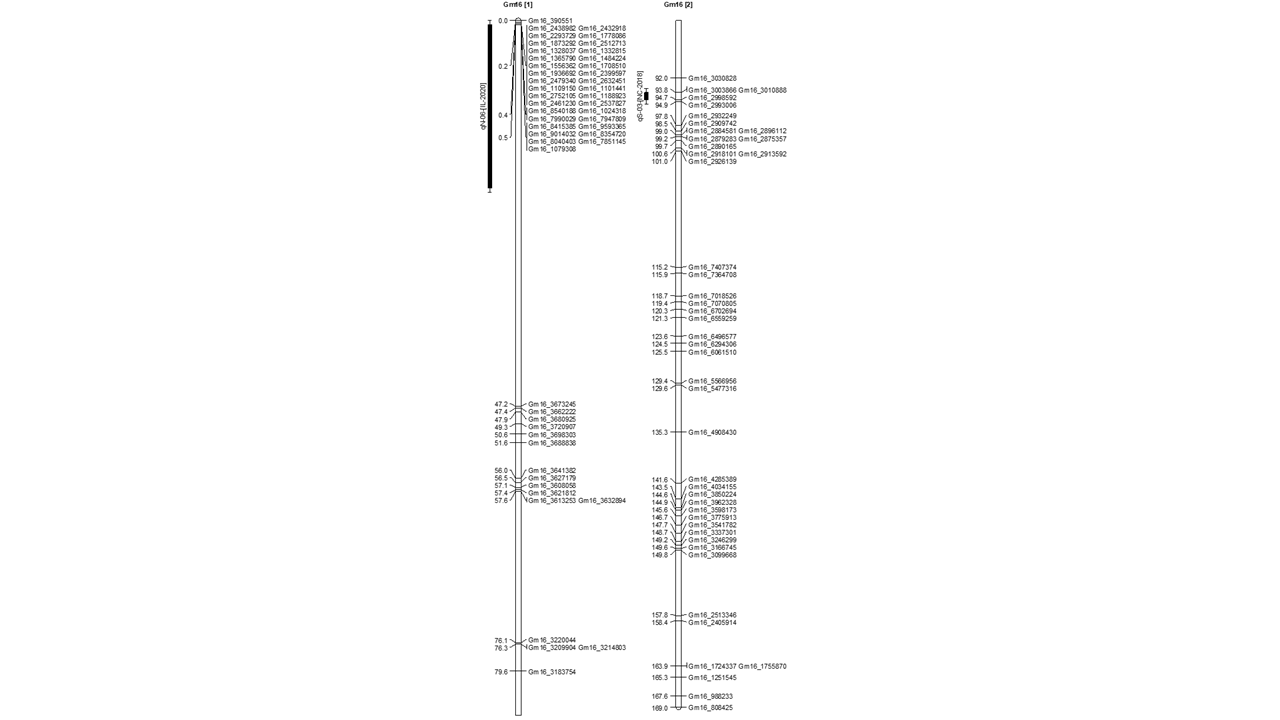

Supplement: S12 Fig — (TIF) [file pone.0331214.s015.TIF]

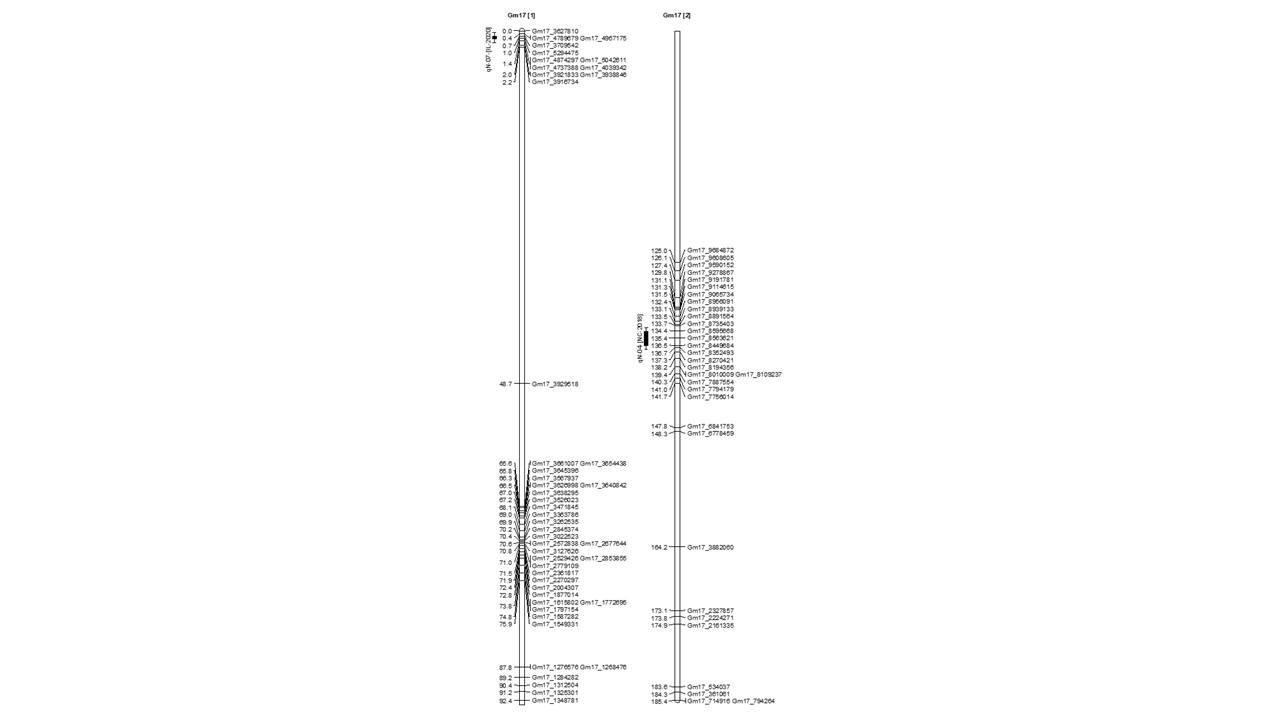

Supplement: S13 Fig — (TIF) [file pone.0331214.s016.TIF]

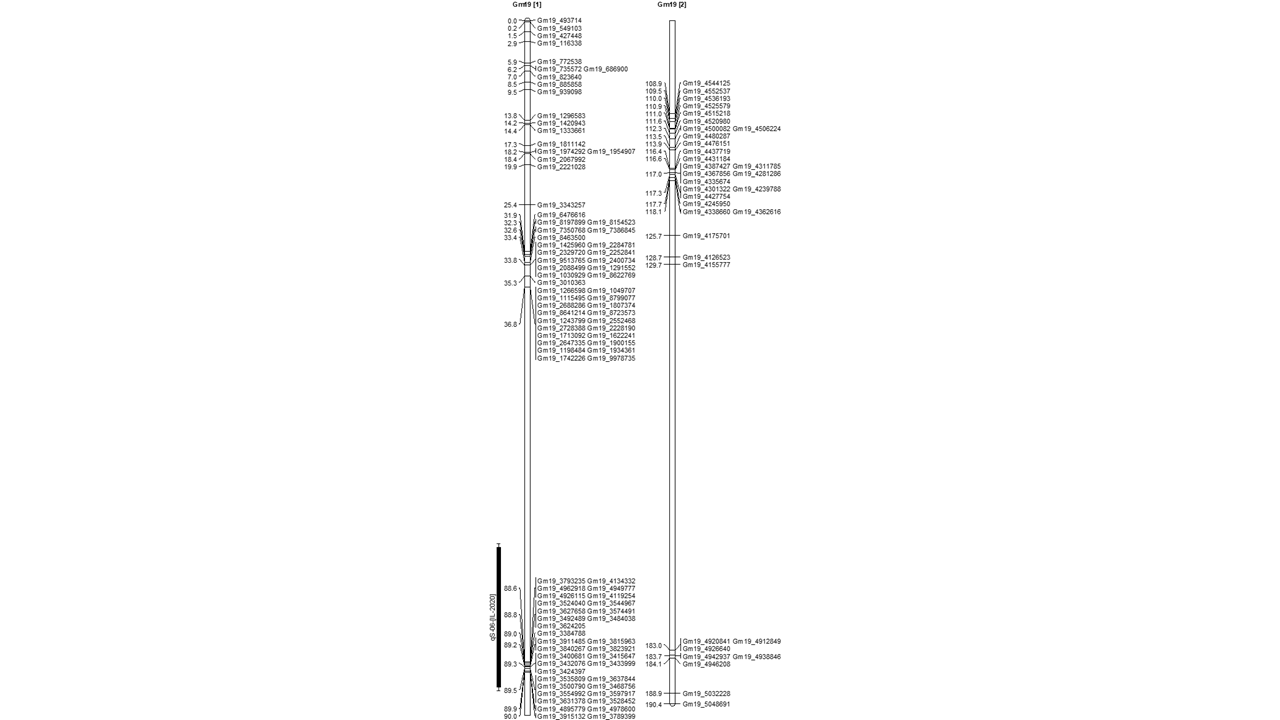

Supplement: S14 Fig — (TIF) [file pone.0331214.s017.TIF]

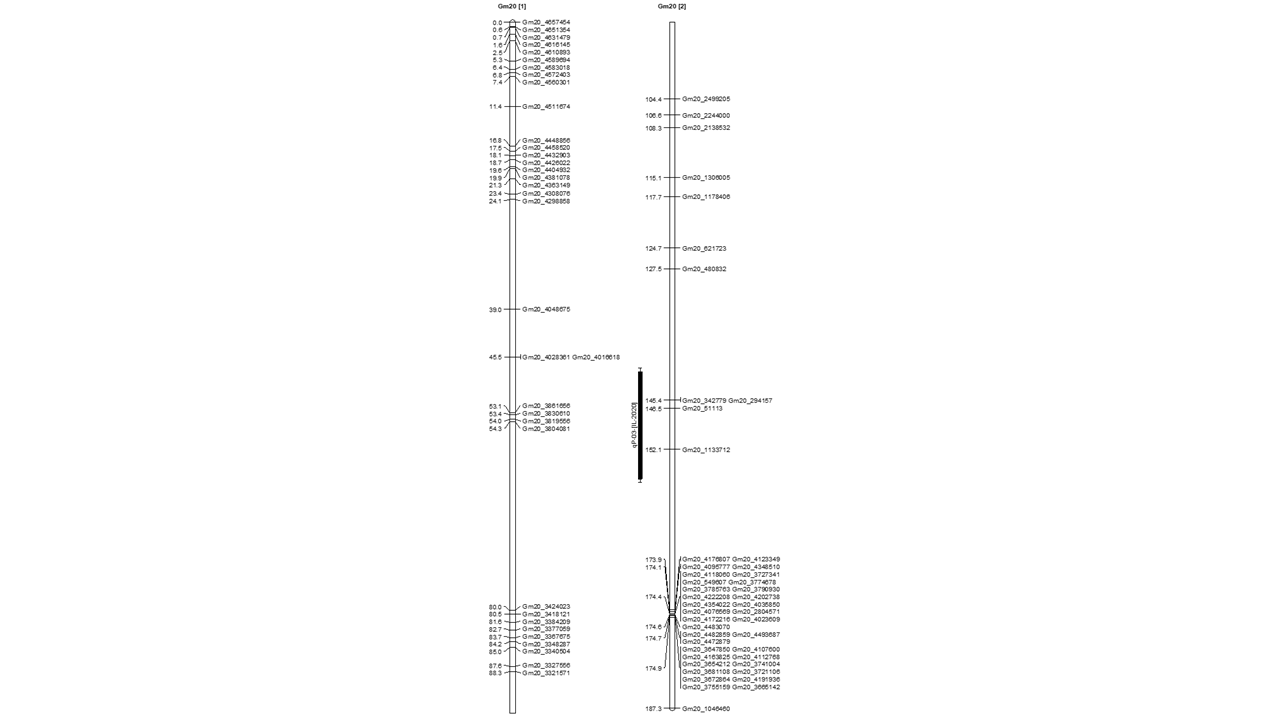

Supplement: S15 Fig — (TIF) [file pone.0331214.s018.TIF]

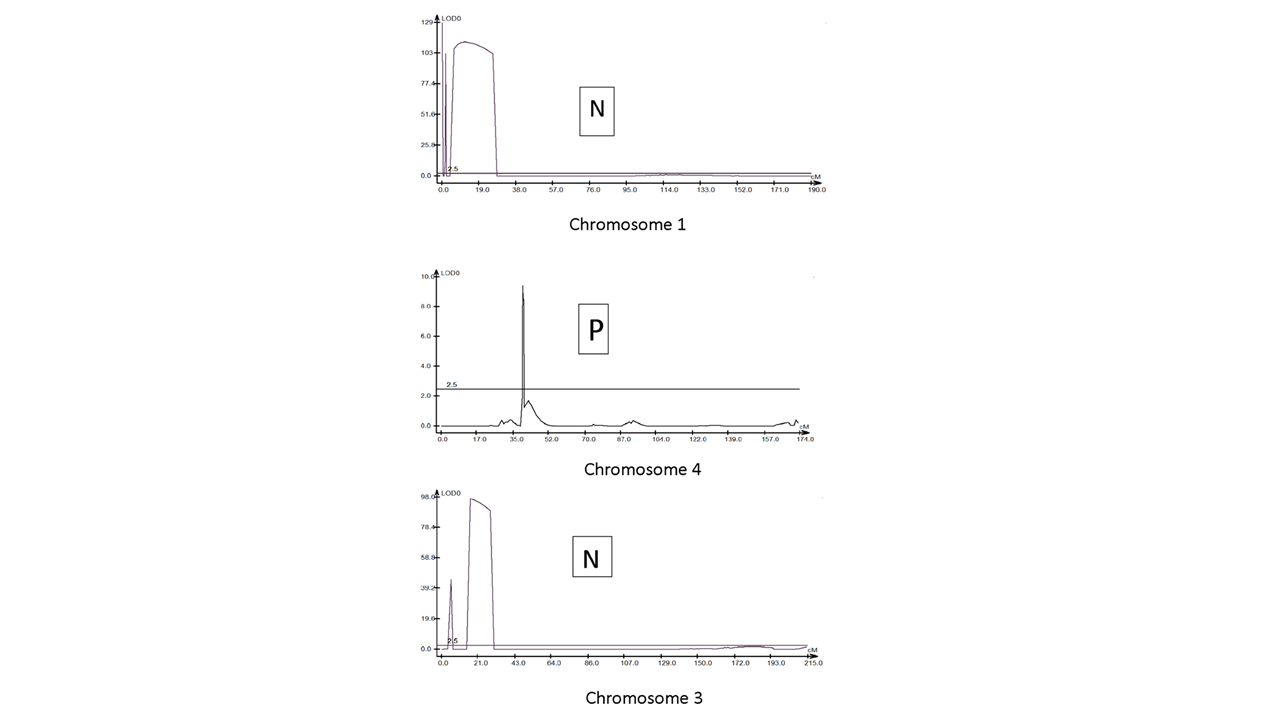

Supplement: S16 Fig — (TIF) [file pone.0331214.s019.TIF]
