# Supplementary material for: Genomic regions and candidate genes associated with seed nitrogen, phosphorus, and sulfur accumulation identified in the soybean ‘Forrest’ by ‘Williams 82’ RIL population
Source: PLoS One. 2025 Sep 3;20(9):e0331214. doi: 10.1371/journal.pone.0331214 (PMC12407463; doi:10.1371/journal.pone.0331214)
Supplement: Attachment — Submitted filename: PONE-D-24-39665with suggestions.pdf [file pone.0331214.s020.pdf]

PLOS ONE

Genomic Regions and Candidate Genes Associated with Seed Nitrogen, Phosphorus, and Sulfur Accumulation Identified in the Soybean ‘Forrest’ by ‘Williams 82’ RIL Population

--Manuscript Draft--

**Manuscript Number:**	PONE-D-24-39665
**Article Type:**	Research Article
**Full Title:**	Genomic Regions and Candidate Genes Associated with Seed Nitrogen, Phosphorus, and Sulfur Accumulation Identified in the Soybean ‘Forrest’ by ‘Williams 82’ RIL Population
**Short Title:**	Genomic Regions and Candidate Genes Associated with soybean Seed nutrients
**Corresponding Author:**	Nacer Bellaloui<br>USDA Agricultural Research Service<br>Stoneville, UNITED STATES OF AMERICA
**Keywords:**	Soybean; RIL; Forrest; Williams 82; linkage map; micronutrients genes; nitrogen, phosphorus, sulfur; SNP, ~~Glycine max~~
**Abstract:**	Nitrogen (N), phosphorus (P), and sulfur (S) are essential nutrients for plant health. Deficiencies in N, P, or S in plants lead to lower seed production and seed quality in grain crops, including soybean seed. Soybean seed is a source of protein, oil, essential amino acids, and minerals. These nutrients are essential for plant health, and maintaining N, P, and S levels in soybean seed is crucial for higher seed nutritional value and amino acids quality. ~~Our previous published research identified QTL and candidates genes related to Fe and Zn soybean seeds. However,~~ there is ~~also~~ limited information on genomic regions (QTL), candidate genes, and molecular markers associated with soybean seed N, P, and S. ~~Therefore, the objective of this research was to identify genomic regions and candidate genes associated with N, P, and S accumulation in soybean seed.~~ Two field experiments were carried out in two locations using a ‘Forrest’ × ‘Williams 82’ recombinant inbred lines (RIL) population. ~~One experiment was conducted in 2018 in Spring Lake, NC; the second experiment was conducted in 2020 in Carbondale, IL.~~ A 306 RIL population and 2075 SNP markers were used to create the genetic map. The results showed a wide range of N, P, and S concentrations in both locations among RIL population ~~lines~~. Based on the broad-sense heritability (H2), 91.7% of seed N concentration variation was due to genetic effects, followed by 48.2% for S seed concentration, and a heritability of close to zero for seed P concentration. Eleven QTL were identified for seed N, 7 QTL for seed P, and 9 QTL for seed S in two locations. All these QTL had a significant linkage to the trait as their LOD ranged from 2.5 to 6.48 in 2018 and from 2.75 to 128.72 in 2020. Two QTL for seed N (qN-02-[IL-2020] on Chr 4, and qN-03-[IL-2020] on Chr 4 were identified at the marker Gm04_4687302-Gm04_7672403 and Gm04_7672403, and their LOD were 45.06 and 96.98, and their contribution to the phenotypic variation were 45.85% and 48.37%, respectively. Except for the seed N, P, and S QTL identified on Chr 16, 11 QTL reported here were not previously identified and therefore are novel. Several functional genes encoding N-, P-, and S-proteins, enzymes, and transporters were identified and located within the QTL interval. To our knowledge, the QTL identified here on Chr 2 and 6 are novel and were not previously identified. Therefore, QTL, genes, and molecular markers discovered in this research will provide breeders with new knowledge and tools for soybe[illegible]elect for optimum seed mineral nutritional qualities. Also, this new knowledge advance our knowledge of physiology and genetics of seed N and S candidate genes for genetic engineering application.
**Order of Authors:**	Nacer Bellaloui
	Jiazheng Yuan
	Dounya Knizia
	Qijian Song, PhD
	Frances Betts

Powered by Editorial Manager® and ProduXion Manager® from Aries Systems Corporation

	Teresa Register
	Earl Williams
	Naoufal Lakhssassi, PhD
	Hamid Mazouz
	Henry T. Nguyen, PhD
	Khalid Meksem, PhD
	Alemu Mengistu, PhD
	My Abdelmajid Kassem
**Additional Information:**	
**Question**	**Response**
**Financial Disclosure**<br><br>Enter a financial disclosure statement that describes the sources of funding for the work included in this submission. Review the submission guidelines for detailed requirements. View published research articles from *PLOS ONE* for specific examples.<br><br>This statement is required for submission and **will appear in the published article** if the submission is accepted. Please make sure it is accurate.<br><br>**Funded studies**<br>Enter a statement with the following details:<br>• Initials of the authors who received each award<br>• Grant numbers awarded to each author<br>• The full name of each funder<br>• URL of each funder website<br>• Did the sponsors or funders play any role in the study design, data collection and analysis, decision to publish, or preparation of the manuscript?<br><br>Did you receive funding for this work?	Yes
Please add funding details.<br>as follow-up to "**Financial Disclosure**<br><br>Enter a financial disclosure statement that describes the sources of funding for the work included in this submission. Review the submission guidelines for detailed	This research was funded by the U.S. Department of Agriculture, Agricultural Research Service Project 6066-21220-016-000D, SIUC, UM, and FSU.

requirements. View published research articles from *PLOS ONE* for specific examples.<br><br>This statement is required for submission and **will appear in the published article** if the submission is accepted. Please make sure it is accurate.<br><br>**Funded studies**<br>Enter a statement with the following details:<br>• Initials of the authors who received each award<br>• Grant numbers awarded to each author<br>• The full name of each funder<br>• URL of each funder website<br>• Did the sponsors or funders play any role in the study design, data collection and analysis, decision to publish, or preparation of the manuscript?<br><br>Did you receive funding for this work?"	
Please select the country of your main research funder (please select carefully as in some cases this is used in fee calculation).<br>as follow-up to "**Financial Disclosure**<br><br>Enter a financial disclosure statement that describes the sources of funding for the work included in this submission. Review the submission guidelines for detailed requirements. View published research articles from *PLOS ONE* for specific examples.<br><br>This statement is required for submission and **will appear in the published article** if the submission is accepted. Please make sure it is accurate.	UNITED STATES - US

Powered by Editorial Manager® and ProduXion Manager® from Aries Systems Corporation

**Funded studies**<br>Enter a statement with the following details:<br>• Initials of the authors who received each award<br>• Grant numbers awarded to each author<br>• The full name of each funder<br>• URL of each funder website<br>• Did the sponsors or funders play any role in the study design, data collection and analysis, decision to publish, or preparation of the manuscript?<br><br>Did you receive funding for this work?"	
**Competing Interests**<br><br>Use the instructions below to enter a competing interest statement for this submission. On behalf of all authors, disclose any competing interests that could be perceived to bias this work—acknowledging all financial support and any other relevant financial or non-financial competing interests.<br><br>This statement is required for submission and **will appear in the published article** if the submission is accepted. Please make sure it is accurate and that any funding sources listed in your Funding Information later in the submission form are also declared in your Financial Disclosure statement.<br><br>View published research articles from *PLOS ONE* for specific examples.	The authors have declared that no competing interests exist.

Powered by Editorial Manager® and ProduXion Manager® from Aries Systems Corporation

**NO authors have competing interests**<br><br>Enter: *The authors have declared that no competing interests exist.*<br><br>**Authors with competing interests**<br><br>Enter competing interest details beginning with this statement:<br><br>*I have read the journal's policy and the authors of this manuscript have the following competing interests: [insert competing interests here]*<br><br>* typeset	
**Ethics Statement**<br><br>Enter an ethics statement for this submission. This statement is required if the study involved:<br><br>• Human participants<br>• Human specimens or tissue<br>• Vertebrate animals or cephalopods<br>• Vertebrate embryos or tissues<br>• Field research<br><br>Write "N/A" if the submission does not require an ethics statement.<br><br>General guidance is provided below. Consult the submission guidelines for detailed instructions. **Make sure that all information entered here is included in the Methods section of the manuscript.**	N/A

**Format for specific study types**<br><br>**Human Subject Research (involving human participants and/or tissue)**<br>• Give the name of the institutional review board or ethics committee that approved the study<br>• Include the approval number and/or a statement indicating approval of this research<br>• Indicate the form of consent obtained (written/oral) or the reason that consent was not obtained (e.g. the data were analyzed anonymously)<br><br>**Animal Research (involving vertebrate animals, embryos or tissues)**<br>• Provide the name of the Institutional Animal Care and Use Committee (IACUC) or other relevant ethics board that reviewed the study protocol, and indicate whether they approved this research or granted a formal waiver of ethical approval<br>• Include an approval number if one was obtained<br>• If the study involved *non-human primates*, add *additional details* about animal welfare and steps taken to ameliorate suffering<br>• If anesthesia, euthanasia, or any kind of animal sacrifice is part of the study, include briefly which substances and/or methods were applied<br><br>**Field Research**<br><br>Include the following details if this study involves the collection of plant, animal, or other materials from a natural setting:<br>• Field permit number<br>• Name of the institution or relevant body that granted permission	
**Data Availability**<br><br>Authors are required to make all data underlying the findings described fully available, without restriction, and from the time of publication. PLOS allows rare exceptions to address legal and ethical concerns. See the PLOS Data Policy and FAQ for detailed information.	No - some restrictions will apply

Powered by Editorial Manager® and ProduXion Manager® from Aries Systems Corporation

A Data Availability Statement describing where the data can be found is required at submission. Your answers to this question constitute the Data Availability Statement and **will be published in the article**, if accepted.<br><br>**Important:** Stating 'data available on request from the author' is not sufficient. If your data are only available upon request, select 'No' for the first question and explain your exceptional situation in the text box.<br><br>Do the authors confirm that all data underlying the findings described in their manuscript are fully available without restriction?	
**Describe where the data may be found in full sentences. If you are copying our sample text, replace any instances of XXX with the appropriate details.**<br><br>• If the data are **held or will be held in a public repository**, include URLs, accession numbers or DOIs. If this information will only be available after acceptance, indicate this by ticking the box below. For example: *All XXX files are available from the XXX database (accession number(s) XXX, XXX.).*<br>• If the data are all contained **within the manuscript and/or Supporting Information files**, enter the following: *All relevant data are within the manuscript and its Supporting Information files.*<br>• If neither of these applies but you are able to provide **details of access elsewhere**, with or without limitations, please do so. For example:<br><br>*Data cannot be shared publicly because of [XXX]. Data are available from the XXX Institutional Data Access / Ethics Committee (contact via XXX) for researchers who meet the criteria for access to confidential data.*<br><br>*The data underlying the results presented in the study are available from (include the name of the third party*	All relevant data are within the manuscript and its Supporting Information files.

and contact information or URL).<br>• This text is appropriate if the data are owned by a third party and authors do not have permission to share the data.<br><br>* typeset	
Additional data availability information:	Tick here if the URLs/accession numbers/DOIs will be available only after acceptance of the manuscript for publication so that we can ensure their inclusion before publication.

Revised Manuscript

Click here to access/download;Manuscript;Bellaloui et al Plos One 2 22 2025 revised.docx

Genomic Regions and Candidate Genes Associated with Seed Nitrogen, Phosphorus, and Sulfur Accumulation Identified in the Soybean 'Forrest' by 'Williams 82' RIL Population

Nacer Bellaloui[1]*, Jiazheng Yuan[2], Dounya Knizia[3,4], Qijian Song[5], Frances Betts[2], Teresa Register[2], Earl Williams[2], Naoufal Lakhssassi[3], Hamid Mazouz[4], Henry T. Nguyen[6], Khalid Meksem[3], Alemu Mengistu[7] and My Abdelmajid Kassem[2]

[1]USDA, Agriculture Research Service, Crop Genetics Research Unit, 141 Experiment Station Road, Stoneville, MS 38776, USA; nacer.bellaloui@usda.gov (N.B.)

[2]Plant Genomics and Biotechnology Laboratory, Department of Biological and Forensic Sciences, Fayetteville State University, Fayetteville, NC 28301, USA; mkassem@uncfsu.edu (M.A.K); jyuan@uncfsu.edu (J.Y.); fbetts@broncos.uncfsu.edu (F.B.); tregist2@broncos.uncfsu.edu (T.R.); ewilli17@broncos.uncfsu.edu (E.W.)

[3]Department of Plant, Soil, and Agricultural Systems, Southern Illinois University, Carbondale, IL 62901, USA; dounya.knizia@siu.edu (D.K.); naoufal.lakhssassi@siu.edu (N.L.); meksem@siu.edu (K.M.)

[4]Laboratoire de Biotechnologies & Valorisation des Bio-Ressources (BioVar), Department de Biology, Faculté des Sciences, Université Moulay Ismail, Meknès 50000, Morocco; h.mazouz@fs-umi.ac.ma (H.M.)

[5]Soybean Genomics and Improvement Laboratory, USDA-ARS, Beltsville, MD 20705, USA; qijian.song@usda.gov (Q.S.)

[6]Division of Plant Science and Technology, University of Missouri, Columbia, MO 65211, USA; nguyenhen-ry@missouri.edu (H.T.N.)

[7]USDA, Agricultural Research Service, Crop Genetics Research Unit, Jackson, TN 38301, USA;
alemu.mengistu@usda.gov (A.M.)

* Correspondence: nacer.bellaloui@usda.gov

Abstract

Nitrogen (N), phosphorus (P), and sulfur (S) are essential nutrients for plant health. Deficiencies in N, P, or S in plants lead to lower seed production and seed quality in grain crops, including soybean seed. Soybean seed is a source of protein, oil, essential amino acids, and minerals. These nutrients ~~components~~ are essential ~~for human and livestock nutrition and~~ for plant health, and maintaining N, P, and S levels in soybean seed is crucial for higher seed nutritional value and amino acids quality. ~~Our previous published research identified QTL and candidates genes related to Fe and Zn soybean seeds. However, there is also~~ l~~L~~imited information ~~is available~~ on genomic regions (QTL), candidate genes, and molecular markers associated with soybean seed ~~the accumulation of these nutrients in soybean seed~~N, P, and S. Therefore, ~~identifying genomic regions, candidate genes, and molecular markers associated with N, P, and S metabolism are important. T~~ ~~the objective of this research was to identify genomic regions and candidate genes associated with N, P, and S accumulation in soybean seed.~~ Two field experiments were carried out ~~conducted~~ in two locations using a ‘Forrest’ × ‘Williams 82’ recombinant inbred lines (RIL) population. ~~The genetic map used in the current research was based on 306 RILs and 2075 SNP markers.~~ ~~One experiment was conducted in 2018 in Spring Lake, NC; the second experiment was conducted in 2020 in Carbondale, IL.~~ ~~The genetic map used in the current research was based~~ A~~on~~ 306 RIL population ~~s~~ and 2075 SNP markers were used to create the genetic map.~~;~~ The results showed a wide range of N, P, and S concentrations in both locations among RIL population lines. Based on the broad-sense heritability (H^2), 91.7% of seed N concentration variation was due to genetic effects, followed by 48.2% for S seed concentration, and a heritability of close to zero for seed P concentration. Eleven QTL were identified for seed N, 7 QTL for seed P, and 9 QTL for seed S in two locations. All these QTL had a significant linkage to the trait as their LOD ranged from 2.5 to 6.48 in 2018 and from 2.75 to 128.72 in 2020. Two QTL for seed N (*qN-02*-[IL-2020] on

Chr 4, and *qN-03*-[IL-2020] on Chr 4 were identified at the marker Gm04_4687302-Gm04_7672403 and Gm04_7672403, and their LOD were 45.06 and 96.98, and their contribution to the phenotypic variation were 45.85% and 48.37%, respectively. Except for the seed N, P, and S QTL identified on Chr 16, 11 QTL reported here were not previously identified and therefore are novel. Several functional genes encoding N-, P-, and S-proteins, enzymes, and transporters were identified and located within the QTL interval. To our knowledge, the QTL identified here on Chr 2 and 6 are novel and were not previously identified. Therefore, QTL, genes, and molecular markers discovered in this research will provide ~~help~~ breeders with new knowledge and tools for soybean select for optimum seed mineral nutritional qualities. Also, this new knowledge ~~These findings also~~ advance our knowledge of physiology and genetics of ~~seed mineral accumulation; and provide new knowledge on~~ seed N and S candidate genes for ~~possible application in~~ genetic engineering application.

Keywords: soybean; RIL; Forrest; Williams 82; linkage map; micronutrients genes; nitrogen, phosphorus, sulfur; SNP, ~~*Glycine max*.~~

1. Introduction

Soybean is a major source of protein, amino acids, oil, fatty acids, and macronutrients, including nitrogen (N), phosphorus (P), and sulfur (S). Nitrogen, P, and S are essential nutrients affecting the growth, development, and crop production. Therefore, maintaining the optimum level of these nutrients in crop seeds is critical to ensure seed health. Nitrogen is essential for productivity [1,2,3], a source of amino acid and protein synthesis, and protein storage [4-9], DNA and RNA, phytohormones, co-enzymes, and involved in several physiological, metabolic, and biochemical reaction [10,11]. Phosphorus is a source of phytic acid

(anti-nutritional component, especially at high level in seeds), and involved in cell membrane structure, function, cell membrane, lipid synthesis, ATP and NADP-H, DNA and RNA, carbohydrate metabolism, and active uptake [10-12]. Like N and P, S is also a critical nutrient and involved in specific amino acids such as S-containing amino acids (cysteine and methionine, two amino acids that are deficient in soybean cultivars, and as a result in protein meal) [13], enzymes and co-enzymes, and essential for N uptake [10-12].

Our literature search showed that gGenomic regions and gene candidates controlling seed N, P, and S accumulation is limited. Searching SoyBase revealed limited molecular markers and QTL associated with root and shoot N, P, and S, were identified, and not in mature soybean seed. (https://www.soybase.org/search/index.php?searchterm=Nitrogen+and+Phosphorus+and+Sulfur&list=bi_parental_qtl_listview) [14]. For example, literature available reported QTL and molecular markers associated with shoot tissue concentrations of macro- and micro-nutrients, including N, P, and S [15]. They were able, in Genome-wide association (GWAS) studies using 31,748 SNPs, to identify several putative loci for macro- and micro-nutrients in soybean shoot, including one QTL for P (*qPHO001*), one QTL for N (*qNIT001*), one QTL for S (*qSUL001*). They suggested that QTL clustering of P, K, Mg, C, N, and S indicated physiological and genetic relationships, and possible similar metabolic processes between these nutrients. A QTL analysis was conducted to understand the genetic mechanism of leaf-related traits [15]. They used RILs of 200 individuals resulted from a cross between the cultivars ‘Nandou 12’ and ‘Jiuyuehuang’. They found 6366 SNPs markers that covered the whole genome of soybean distributed on 20 chromosomes and spanned 2818.67cM with an average interval of 0.44cM between adjacent markers. They were able to identify 19 QTL and 3 candidate genes associated to leaf-related traits [15].

Further, previous research indicated that most of the genetic mapping for plant nutrition was conducted on leaves, roots, or shoot [3,15,16], and not on nutrients accumulation in mature seeds [3, 17-18]. Others were able to associate genotypic differences in soybean seed nitrogen accumulation with genomic regions controlling nitrogen accumulation in soybean during R5, R6, and R7 growth stages [19], but not at R8 (complete seed maturity stage) ~~as in our current research~~. They used a population of 101 F6:8 recombinant inbred lines resulted from a cross of N87-984-16 x TN93-99. They found several QTL on chr 2, 7, 8, 14, 15, 18. They found that the phenotypic variation explained 5 to 11.6%. Also, others [16], working on 184 recombinant inbred lines resulted from Kefeng No. 1 and Nanong 1138-2 soybean varieties, were able to identify QTL associated with P deficiency tolerance in leaves, roots, and shoots. They identified 7 QTL mapped on two chromosomes ~~and~~ associated with weight of fresh shoot, phosphorus contents in leaf and in root. Other associated with P in soybean shoot were reported elsewhere [20-22]. Further, using 92 F5:7 soybean RILs~~recombinant inbred lines~~, derived from a cross between MD 96-5722 and Spencer using 5,376 SNP markers, they were able to identify QTL related to seed N, P, and S [23]. They were able to detect N QTL (*qNIT001*) on Chr 16, ~~at the marker interval ss248983974-ss248977568, with LOD of 9.85 and with a contribution to phenotypic variance of 94% and a positive additive effect of 3.25. In addition, they identified~~ P QTL (*qPHO001*) on Chr16, ~~at marker interval ss248983974-ss248977568 with LOD of 9.49, and a contribution to phenotypic variance of 94% and a positive additive effect of 0.25.~~ Sulfur QTL (*q SUL001*) on ~~Chr16 was also identified at the marker interval of ss248983974-ss248977568 with LOD peak of 9.73 wit[illegible]contribution to the phenotypic variation of 94% and positive additive effects of 0.15~~ [23]. A QTL analysis was performed to understand the genetic mechanism of leaf-related traits [15]. They used RILs of 200 individuals resulted from a cross between the cultivars 'Nandou 12' and 'Jiuyuehuang'. They found 6366 SNPs markers that covered the whole genome of soybean distributed on 20 chromosomes and spanned

2818.67cM with an average interval of 0.44cM between adjacent markers. They were able to identify 19 QTL and 3 gene candidates associated to leaf-related traits [15].

Based on the above literature, very limited information is available on soybean seed N, P, and S accumulation QTL. ~~T~~Our previous published research [30] identified QTL and candidates genes related to Fe and Zn soybean seeds. However, ~~herefore,~~ the current research was aimed at identifying QTL and associated genes for seed N, P, and S accumulation in a RIL population (total of 306 lines), using 5405 SNPs markers.

2. Materials and Methods

2.1 Plant Material and Growth Conditions

A ~~A ‘Forrest’ × ‘Williams 82’~~ RIL population was developed (‘Forrest’ × ‘Williams 82’) for genetic mapping [24]. ~~Briefly, the cultivar~~ ‘Forrest’ was created ~~developed~~ from a cross between ~~the cross of~~ ‘Dyer’ and ‘Bragg’, developed by USDA [25], and ~~. The cultivar~~ ‘Williams 82’ ~~was developed~~ from a cross of ‘Williams’ and ‘Kingwa’ [26]. ~~The genomes of the soybean cultivars are duplicated polyploid genomes with highly conserved gene-rich regions [27]. Although, originally, the ‘Forrest’ × ‘Williams 82’ RIL population was developed with more than 1000 RILs [27],~~ T~~t~~he genetic map ~~used in the current research~~ was based on 306 RILs and 2075 SNP markers [27,28]. Two field experiments were conducted; ~~We have conducted two experiments;~~ one ~~on a farm~~ in Spring Lake, NC (35.17◦ N, 78.97◦ W) in 2018; the second ~~experiment was conducted on a farm~~ in Carbondale, IL (37◦ N, 89◦ W) in 2020. ~~Seeds of parents and RILs~~ Seeds were planted on a 75 cm row-spacing, and ~~.~~ t~~T~~he growth conditions and field management were conducted as previously reported by others [27,28]. ~~Both seeds of parents and RILs were included in both experiments~~

~~[27]. Toxicity or deficiency symptoms of N, S, or P were not observed on soybean during its growth or maturity.~~ Our previous published research ~~Initially, our breeding goal was to~~ mapped QTL for soybean seed protein, oil, isoflavone, and amino acids, and showed that ~~. Therefore,~~ the parents (Williams 82 and Forrest) were contrasting in seed protein, oil, isoflavones, and some amino acids~~, as reported elsewhere~~ [28,29,30]. In the current experiment, ~~mineral analysis of the current~~ RILs~~/progeny~~ showed a wide range of N, S, and P, and some RILs ~~/progeny~~ showed even higher concentrations of N, S, and P than the two parents. This ~~(this~~ means that RIL population showed Transgressive Segregation where the progeny shows more genetic variation and variation in gene expression than their parents~~)~~. ~~This gave us an opportunity to have identified significant QTL associated with N, S, and P.~~ Also, this indicated that ~~In addition,~~ N, S, and P content trait in soybean is~~reflects~~ a complex polygenic trait, as previously reported [29]~~(K)~~, where QTL mapping for different seed nutrients using various mapping biparental populations. Therefore, QTL associated with N, S, and P were pursued.

2.2 Analysis for Seed N and S

The concentrations of N and S in the matured seed were determined by using ground and dried seeds using a Laboratory Mill 3600 (Perten, Springfield, IL USA). Nitrogen and S concentrations measurements in mature seeds were conducted ~~by~~ using a 0.25 g ground-dried sample, and were~~was~~ combusted in an oxygen atmosphere at 1350 ºC, converting elemental N and S into N_2 and SO_2, respectively. Gases of N_2 and SO_2 were then passed through infrared cells and N and S were measured by an elemental analyzer using thermal conductivity cells (LECOCNS-2000 elemental analyzer, LECO Corporation, St. Joseph, MI USA) as previously detailed [31,32].

2.3 Phosphorus Measurement

Mature seeds at R8 were collected and analyzed for P concentrations using spectrophotometer. The concentration of P was determined by the yellow phosphor-vanado-molybdate complex as detailed elsewhere [33]. In short, a sample of 2 g of a dried ground seed was ashed, and then, 10 ml of 6 M HCl was added and placed in a water bath to bring the solution to dryness. Then, 2 ml of 36% v/v HCl were added, and the sample was boiled. The samples were boiled again by adding 10 ml of distilled water. The sample, then was transferred to a 50-mL volumetric flask, diluted to 50 mL with distilled water, filtered, and a volume of 2 ml of filtrate was first discarded and the rest was kept for P analysis. A volume of 5 ml of 5 M HCl and 5 ml of ammonium molybdate–ammonium metavanadate reagent were added to the filtrate, and the mixture was diluted with distilled water to 50 ml. Ammonium molybdate–ammonium metavanadate was prepared by dissolving 25 g of ammonium molybdate and 1.25 g of ammonium metavanadate in 500 ml of distilled water. Dihydrogen orthophosphates was used to prepare a P standard curve (0–50 μg/ml of P). A Beckman Coulter DU 800 spectrophotometer was used to measure the P concentrations at 400 nm.

2.4 DNA Isolation, SNP Genotyping, and Genetic Map Construction

Genomic DNA of the RIL and ~~population lines and their~~the parents were extracted according to others and our previous published work ~~as described in detail in our published work~~ [27,28,30,~~27,~~34]. ~~Briefly, a standard cetyltrimethyl ammonium bromide (CTAB) method with minor modifications [34] was used to extract DNA. The DNA concentration was quantified by a spectrophotometer (NanoDrop Technologies Inc., Centreville, DE, USA). A concentration of 50 ng/μL was used for genotyping. Genotyping was conducted in the Soybean Genomics and Improvement Laboratory, USDA-ARS, Beltsville, MD, USA, using SNPs and the Illumina Infinium SoySNP6K BeadChips (Illumina, Inc. San Diego, CA, USA)~~Genotyping was conducted in the Soybean Genomics and Improvement Laboratory, USDA-ARS, Beltsville, MD, USA, as

detailed by others and by our previous published work [27,28,30,35].~~ SNP alleles were called using GenomeStudio Genotyping Module 2.0 (Illumina, Inc. San Diego, CA, USA). JoinMap 4.0 [34] was used to construct the genetic linkage map.~~ A ~~A LOD score~~ threshold of 2.5 for LOD, -and a maximum genetic distance of 50 cM to group markers were used. The linkage groups were assigned to corresponding soybean chromosomes as described in Soy-Base [36,37].

2.5 N, S, and P QTL Detection, Candidate Genes, and Statistics

Detection of QTL and statistical analysis were performed as previously described [27,28]; ~~. Briefly,~~ the broad sense heritability (H^2) analysis from two-way ANOVA was performed using the equation: $H^2 = \sigma G2/[\sigma G2 + (\sigma GE2/e) + (\sigma e2/re)]$ s described in details by others and by in our previous published work (27,28,30, 38)~~where σG2 (variance of genetic factor), σGE2 (variance of genotype-environment interactions), and σe2 (variance of random effect) were calculated with e (number of environment) and r normalization [38].~~. The significance level was conducted using R package car (type II Wald chi-square tests) (R Software, accessed on June 15, 2023) [39]. To identify QTL for seed N, S, and P concentrations in RIL population, we used Composite Interval Mapping (CIM) methods of Win-QTL Cartographer 2.5 (Windows QTL Cartographer 2.5. Department of Statistics, North Carolina State University, Raleigh, NC; http://statgen.ncsu.edu/qtlcart/WQTLCart.htm) [40]. The default parameters of WinQTL Cartographer were selected (Model 6, 1 cM step size, 10 cM window size, 5 control markers, and 1,000 permutations threshold) [40]. Chromosomes were drawn using MapChart 2.2[41]. Designation of QTL in the two years were done following the approach used by others [28,41. The candidate genes, within identified QTL for N, P, and S content in soybean seeds, were annotated using SoyBase Genome Browser (glyma.Wm82.gnm4). Analysis of Means procedure ~~(maximum and minimum values, and SE)~~ was~~were~~ conducted using Proc

Means in SAS. Correlations were conducted by SAS using PROC REG (SAS, Statistical Analysis Systems, Cary, NC, USA, 2002–2012) [42].

3. Results

A wide range of N, P, and S concentrations were observed in both locations (**Table 1; Figures 1 and 2**). Analysis of variance (ANOVA) showed that year had a significant effects on N (F=476.8; P=0.0001), P (F=32.8; P=0.0001), and S (F=386.7; P=0.0001). Also, line had a significant effect on N (F=29.3; P=0.0001), S (F=1.4; P=0.008), but not significant for P (F=0.9; P=81). A 91.7% (H^2) of seed N trait variation is due to genetics, followed by 48.2% for S seed concentration trait, and finally an inheritance of close to zero for seed P concentration trait (**Table 2**). The low heritability of P indicated that almost all of the variability in this trait is due to environmental factors, and negligeable/very little effects is due to genetic differences. Except for the correlation between N and P in 2018, correlations between nutrients in two years and at both locations were all positive (**Figures 3 and 4**).

Table 1. Statistical components of soybean seed N (mg/kg), P (mg/kg), and S (mg/kg) in ‘Forrest’ by ‘Williams 82’ recombinant inbred soybean lines (RILs) population in 2018 NC and 2020 in IL.

			**2018**					
**Trait**	**Mean**	**Minimum**	**Maximum**	**Median**	**CV (%)**	**SE**	**Skewness**	**Kurtosis**
Nitrogen	63.3	54.7	69.4	63.4	3.91	0.17	-0.36	3.51
Phosphorus	6.2	4.8	7.84	6.30	9.77	0.04	-0.19	2.19
Sulfur	2.7	1.95	3.57	2.74	10.14	0.02	-0.01	2.3

			2020					
**Trait**	**Mean**	**Minimum**	**Maximum**	**Median**	**CV (%)**	**SE**	**Skewness**	**Kurtosis**
Nitrogen	62.57	52.6	94.5	59.10	6.94	0.07	1.73	4.34
Phosphorus	5.96	4.26	7.74	5.93	8.17	0.03	0.27	3.89
Sulfur	3.25	2.57	4.15	3.22	7.61	0.01	0.4	3.29

Table 2. Statistical components (Sum Square and Mean Square) and broad-sense heritability (H^2) for soybean seed N, P, and S in Forrest' by 'Williams 82' recombinant inbred soybean lines (RILs) population across two years (2018 and 2020).

Nitrogen

	**Sum Square**	**Mean Square**	**H^2**
Line	35209	117.36	0.917
Year	1869	1869.44	
Line:Year	1762	9.68	

Phosphorus

	**Sum Square**	**Mean Square**	**H^2**
Line	85.934	0.2864	-0.002
Year	6.616	6.616	
Line:Year	51.251	0.2871	

Sulfur

	Sum Square	Mean Square	H^2
Line	32.938	0.1098	0.482
Year	20.933	20.9334	
Line:Year	9.634	0.0529	

Figure 1. Frequency distribution for seed N (Top), P (Middle), and S (Bottom) in 2018 in the ‘Forrest’ by ‘Williams 82’ recombinant inbred soybean lines (RILs) population in soybean. Gaps that exist in any distribution graph indicate there is zero line in that range.

Figure 2. Frequency distribution for seed N (top), P (middle), and S (bottom) in 2020 in the ‘Forrest’ by ‘Williams 82’ recombinant inbred soybean lines (RILs) population in soybean. Gaps that exist in any distribution graph indicate there is zero line in that range.

Figure 3. Patterns of correlation between seed N vs. P (top), N vs. S (middle), and P vs. S (bottom) in 2018 in the ‘Forrest’ by ‘Williams 82’ recombinant inbred soybean lines (RILs) population in soybean.

Figure 4. Patterns of correlation between seed N vs. P (top), N vs. S (middle), and P vs. S (bottom) in 2020 in the 'Forrest' by 'Williams 82' recombinant inbred soybean lines (RILs) population in soybean.

A total of 11 QTL were detected for seed N on 9 chromosomes (Chr), 7 QTL for seed P on 7 Chr, and 9 QTL for seed S on 7 Chr in two locations (Spring Lake, NC in 2018; and Carbondale, IL in 2020) (**Table 3; Figure S1-S15)**.

Table 3. QTLs that control seed nitrogen (N), phosphorus (P), and sulfur (S) contents in two environments over two years (E1 and E2). E1: Spring Lake, NC (2018) and E2: Carbondale, IL (2020). Only QTL with LOD scores > 2.5 and identified by composite interval mapping (CIM) method of QTL Cartographer (Wang et al., 2012) are reported. Chr= chromosome; Position= The peak position of the significant QTL; LOD= Logarithm of the odds; R^2 =Percentage of variation explained by each identified QTL; Add. Eff.= Additive effect; a negative value indicates that the Forrest allele increased the trait value.

E1: Spring Lake, NC (2018)

**Trait**	**QTL**	**Chr.**	**Marker**	**Pos. (cM)**	**LOD**	**R^2**	**Add. Eff.**
N	*qN-01*-[NC-2018]	2	Gm02_5141136-Gm02_4938821	137.1-295.8	4.51	8.19	1.7601
	qN-02-[NC-2018]	7	Gm07_4292342	8.1-10.1	2.50	4.44	0.5294
	qN-03-[NC-2018]	15	Gm15_1142117-Gm15_1149627	140.2-140.8	2.72	4.54	0.5441
	qN-04-[NC-2018]	17	Gm17_8449684	134.5-136.5	2.94	5.29	0.5958
P	*qP-01*-[NC-2018]	4	Gm04_7672403	6.5-8.5	2.54	4.15	0.3114
	qP-02-[NC-2018]	5	Gm05_1744708-Gm05_1705841	127.9-134.4	3.34	5.54	0.1805

	qP-03-[NC-2018]	12	Gm12_975837-Gm12_1632399	178.7-189.3	6.48	11.10	0.2527
	qP-04-[NC-2018]	13	Gm13_2748576	0.5-4.5	2.51	9.07	0.3739
S	*qS-01*-[NC-2018]	2	Gm02_9925870-Gm02_7987834	146.2-164.5	3.57	5.86	-0.0815
	qS-02-[NC-2018]	5	Gm05_3273418	33.1	2.50	3.89	-0.1583
	qS-03-[NC-2018]	16	Gm16_3010888-Gm16_2998592	93.8-94.7	3.41	5.59	-0.0667

E2: Carbondale, IL (2020)

**Trait**	**QTL**	**Chr.**	**Marker**	**Pos. (cM)**	**LOD**	**R^2**	**Add. Eff.**
N	*qN-01*-[IL-2020]	1	Gm01_3504836-Gm01_3466825	0.1-2.1	128.72	78.52	14.73
	qN-02-[IL-2020]	4	Gm04_4687302-Gm04_7672403	1.4-5.4	45.06	45.85	14.09
	qN-03-[IL-2020]	4	Gm04_7672403	16.5-28.5	96.98	48.37	14.91
	qN-04-[IL-2020]	6	Gm06_1737718-Gm06_5014399	30.7-58.4	25.53	37.57	-11.13
	qN-05-[IL-2020]	10	Gm10_621706-Gm10_6020959	196.1-202.1	47.88	42.05	-13.20

	qN-06-[IL-2020]	16	Gm16_7947809-Gm16_1079308	0.4-20.5	34.57	38.97	-11.13
	qN-07-[IL-2020]	17	Gm17_3709542-Gm17_5294475	0.7-1.1	7.56	7.14	12.04
P	*qP-01*-[IL-2020]	2	Gm02_5141136-Gm02_1020061	137.1-139.8	3.51	4.26	-0.18
	qP-02-[IL-2020]	3	Gm03_4469376-Gm03_4447541	39.3-40.1	9.42	12.17	0.55
	qP-03-[IL-2020]	20	Gm20_480832-Gm20_1133712	141.5-156.1	4.12	5.34	0.13
S	*qS-01*-[IL-2020]	2	Gm02_1084314-Gm02_9925870	138.6-144.2	6.32	7.84	-0.13
	qS-02-[IL-2020]	4	Gm04_4687302-Gm04_7672403	1.4-5.4	2.75	11.51	0.10
	qS-03-[IL-2020]	4	Gm04_4388529-Gm04_4417048	165.3-168.5	3.84	4.51	0.05
	qS-04-[IL-2020]	7	Gm07_1285811-Gm07_1045167	148.6-152.2	3.82	4.49	0.05
	qS-05-[IL-2020]	10	Gm10_4842697-Gm10_4777478	11.1-16.7	3.25	3.76	0.05
	qS-06-[IL-2020]	19	Gm19_9978735-Gm19_3789399	72.8-92.1	6.64	7.93	0.22

In 2018, the QTL for seed N explained 4.15% to 8.19% of the phenotypic variation; for seed P explained 4.15% to 11.10%; for seed S, the QTL explained 3.89% to 5.86%. In 2020, the QTL for seed N explained 7.14 to 78.52%; for seed P, the QTL explained 4.26 to 12.17%; for seed S, the QTL explained 7.84 to 11.51% of phenotypic variation. All these QTL had a significant linkage to the trait as their LOD ranged from 2.5 to 6.48 in 2018 and from 2.75 to 128.72 in 2020 (**Table 3; Figure S1-S15**).

In 2018, and for seed N QTL, the highest LOD (4.51) was observed for N QTL *qN-01*-[NC-2018] on Chr 2 with the marker Gm02_5141136-Gm02_4938821 at marker intervals of 137.1-295.8 cM, and a contribution to the phenotypic variation of 8.19%. For P QTL, the highest LOD (6.48) was observed for QTL *qP-03*-[NC-2018] with the marker Gm12_975837-Gm12_1632399, at 178.7-189.3 cM, with a contribution of 11.10%. For S QTL, the highest LOD (3.57) was recoded for QTL *qS-01*-[NC-2018] on Chr 2 with a marker Gm02_9925870-Gm02_7987834 at 146.2-164.5 cM with a contribution of the trait of 5.86%.

In 2020, the LOD for seed N QTL was highly significant, but the highest (LOD of 128.72) were recorded for QTL *qN-01*-[IL-2020] on Chr 1 with the marker Gm01_3504836-Gm01_3466825 at 0.1-2.1 cM (**Table 3**). The second highest LOD was recoded for seed N QTL *qN-04*-[IL-2020] with LOD of 96.98 (**Table 3; Figure S1-S15**). For seed P QTL, the highest LOD (9.42) was for QTL qP-02-[IL-2020] at marker Gm03_4469376-Gm03_4447541 and at the position 39.3-40.1 cM. For seed S QTL, the highest two LOD were recoded for QTL *qS-01*-[IL-2020] on Chr 2 with LOD 6.32, and at marker Gm02_1084314-Gm02_9925870 at position 138.6-144.2 cm; and for QTL *qS-06*-[IL-2020] on Chr 19 with LOD of 6.64 at marker Gm19_9978735-Gm19_3789399 and at position of 72.8-92.1cM. The contribution of the QTL *qN-01*-[NC-2018] on Chr 2, with the highest LOD (6.48), to the phenotypic variation was 8.19%; for seed P, the

highest contribution (11.10%) to the phenotypic variation was recorded on QTL *qP-03*-[NC-2018] on Chr 12; for S, the highest contribution (3.57) to the phenotypic variation was observed on QTL *qS-06*-[IL-2020] on Chr 19.

In 2020, and with the except for QTL *qN-07*-[IL-2020], the contribution of the rest of seed N QTL ranged from 37.57 to 78.52%, with the two highest were recorded on QTL *qN-01*-[IL-2020] on Chr 1with LOD of 128.72 % with the marker Gm01_3504836-Gm01_3466825 at 0.1-2.1 cM; and QTL *qN-02*-[IL-2020] on Chr 4 with 96.98% with the marker Gm04_7672403 at position 16.5-28.5 cM. The highest contribution to the phenotypic variation for seed P QTL *qP-02*-[IL-2020] was recorded on QTL with 12.17 %. For seed S, the highest contribution to the phenotypic variation was recorded for QTL *qS-02*-[IL-2020] on Chr 4 with 11.51%. In 2018, positive additive effects for seed N QTL and P seed QTL were observed; however, negative additive effects for S seed QTL (**Table 3**). A positive and negative additive effects for seed N QTL, P QTL and S QTL. It is clear the additive effect, either positive or negative were greater in 2020 (**Table 3)**.

Two QTL for seed N (*qN-02*-[IL-2020] on Chr 4; and *qN-03*-[IL-2020] on Chr 4 were identified at the marker Gm04_4687302-Gm04_7672403 and Gm04_7672403, respectively, and respectively at peak of 1.4-5.4 cM and 16.5-28.5 cM. Their LOD were highly significant 45.06, 96.98 (**Table 3; Figure S16**), and their contribution to the phenotypic variation were 45.85% and 48.37%, respectively for (qN-02-[IL-2020]. The additive effects were both positive and they were counted for 14.09 and 14.91, respectively for *qN-02*-[IL-2020] and *qN-03*-[IL-2020]. Similar observation was noticed for seed S trait for *qS-02*-[IL-2020] and *qS-03*-[IL-2020] on Chr

5 Gm04_4687302-Gm04_7672403 and Gm04_4388529-Gm04_4417048 at 1.4-5.4cM and 165.3-168.5 cM, respectively. Their LOD, respectively for *qS-02*-[IL-2020] and *qS-03*-[IL-2020], were 2.75 and 3.84 (both were significant), with a contribution to phenotypic variance of 11.51 and 4.5, and additive effects of 1 0.10 and 0.05, much lower than those of *qN-02*-[IL-2020] and *qN-03*-[IL-2020]. Using SoyBase Genome Browser (glyma.Wm82.gnm4), several functional genes encode N-, P-, and S-proteins, enzymes and transporters were identified and located within the QTL intervals (**Table 4 and 5**).

Table 4. QTL and candidate genes for seed N, P, and S accumulation in the Forrest' by 'Williams 82' recombinant inbred soybean lines (RILs) population (RILs) population in soybean in two environments [(Spring Lake, NC (2018); and Carbondale, IL (2020)]. Only QTL with LOD scores ≥ 2.5 and identified by composite interval mapping (CIM) method of QTL Cartographer (Wang et al., 2012), were reported. Candidate genes, controlling N, P, and S accumulation associated with previously reported QTLs using SoyBase Genome Browser, are presented.

**Trait**	**Environment**	**Chr**	**Genomic Interval**	**Candidate Genes**
	qN-01-[NC-2018]	2	Gm02_5141136-Gm02_4938821	Glyma.02G043700/Glyma.02G041900
	qN-02-[NC-2018]	7	Gm07_4292342<br>Gm07_4292342	Glyma.07G045900
	qN-03-[NC-2018]	15	Gm15_1142117-Gm15_1149627	Glyma.15G015600

Formatted Table

**N**	*qN-04*-[NC-2018]	17	Gm17_8449684	Glyma.17G110701/Glyma.17G106800
	qN-01-[IL-2020]	1	Gm01_3504836-Gm01_3466825	Glyma.01G033700/Glyma.01G033800/ Glyma.01G033900/Glyma.01G034300
	qN-02-[IL-2020]	4	Gm04_4687302-Gm04_7672403	Glyma.04G058600/Glyma.04G060600/ Glyma.04G073800
	qN-03-[IL-2020]	4	Gm04_7672403	Glyma.04G073800/Glyma.04G080700
	qN-04-[IL-2020]	6	Gm06_1737718-Gm06_5014399	Glyma.06G055100
	qN-05-[IL-2020]	10	Gm10_621706-Gm10_6020959	Glyma.10G030800
	qN-06-[IL-2020]	16	Gm16_7947809-Gm16_1079308	Glyma.16G041200

	qN-07-[IL-2020]	17	Gm17_3709542-Gm17_5294475	Glyma.17G067200/Glyma.17G067301
	qP-01-[NC-2018]	4	Gm04_7672403	Glyma.04G088000
	qP-02-[NC-2018]	5	Gm05_1744708-Gm05_1705841	Glyma.05G020500
**P**	*qP-03*-[NC-2018]	12	Gm12_975837-Gm12_1632399	Glyma.12G014400/Glyma.12G019600
	qP-04-[NC-2018]	13	Gm13_2748576	Glyma.13G004900
	qP-01-[IL-2020]	2	Gm02_5141136-Gm02_1020061	Glyma.02G054800
	qP-02-[IL-2020]	3	Gm03_4469376-Gm03_4447541	Glyma.03G043675

	qP-03-[IL-2020]	20	Gm20_480832-Gm20_1133712	Glyma.20G011900/Glyma.20G018000
	qS-01-[NC-2018]	2	Gm02_9925870-Gm02_7987834	Glyma.02G095500
**S**	*qS-02*-[NC-2018]	5	Gm05_3273418	Glyma.05G038100
	qS-03-[NC-2018]	16	Gm16_3010888-Gm16_2998592	Glyma.16G030800
	qS-01-[IL-2020]	2	Gm02_1084314-Gm02_9925870	Glyma.02G095500
	qS-02-[IL-2020]	4	Gm04_4687302-Gm04_7672403	Glyma.04G080300

Table 5. Candidate genes and their functional annotation for seed N, P, and S accumulation in the Forrest' by 'Williams 82' recombinant inbred soybean lines (RILs) population in soybean in two environments [(Spring Lake, NC (2018); and Carbondale, IL (2020)]. Only QTL with LOD scores ≥ 2.5 and identified by composite interval mapping (CIM) method of QTL Cartographer (Wang et al., 2012) are reported. Candidate genes, controlling N, P, and S accumulation associated with previously reported QTLs using SoyBase Genome Browser, are presented.

**Candidate Genes**	**Reference Genome**	**Functional annotation**
Glyma.02G043700/Glyma.02G041900	Glyma4.0	Ammonium transporter 2/phosphoserine aminotransferase
Glyma.07G045900	Glyma4.0	Alanine aminotransferase 2
Glyma.15G015600	Glyma4.0	Nodulin MtN21 /EamA-like transporter family protein

Formatted Table

Glyma.17G110701/Glyma.17G106800	Glyma4.0	Nodulin-like / Major Facilitator Superfamily protein/<br>glutamine-dependent NAD(+) synthetase
Glyma.01G033700/Glyma.01G033800/Glyma.01G033900/Glyma.01G034300	Glyma4.0	Nodulin MtN21 /EamA-like transporter family/<br>YLS7-like associated with nitrogen metabolism
Glyma.04G058600/Glyma.04G060600/Glyma.04G073800	Glyma4.0	NAD(P)-binding Rossmann-fold superfamily protein/<br>early nodulin-like protein 15/N-acetyl-l-glutamate kinase
Glyma.04G073800/Glyma.04G080700	Glyma4.0	Early nodulin-like protein 15/N-acetyl-l-glutamate kinase/<br>aspartate aminotransferase 3
Glyma.06G055100	Glyma4.0	Nitrilase-like protein 1
Glyma.10G030800	Glyma4.0	Ammonium transmembrane transporter

Glyma.16G041200	Glyma4.0	Glutamate dehydrogenase 1
Glyma.17G067200/Glyma.17G067301	Glyma4.0	Glutamate receptor/glutamate receptor 2.8-like protein
Glyma.04G088000	Glyma4.0	Inositol-tetrakisphosphate 1-kinase 2-like isoform X1
Glyma.05G020500	Glyma4.0	Probable glycerophosphoryl diester phosphodiesterase 3-like protein
Glyma.12G014400/Glyma.12G019600	Glyma4.0	Nucleotide-diphospho-sugar transferase superfamily protein/putative phosphatidylinositol N-acetylglucosaminyltransferase subunit C-like isoform X1
Glyma.13G004900	Glyma4.0	Choline-phosphate cytidylyltransferase
Glyma.02G054800	Glyma4.0	1-(5-phosphoribosyl)-5-[(5-phosphoribosylamino)methylideneamino] imidazole-4-carboxamide isomerase

Glyma.03G043675	Glyma4.0	Protein phosphatase 2C family protein;
Glyma.20G011900/Glyma.20G018000	Glyma4.0	Purple acid phosphatase 17/Phosphoglucomutase/phosphomannomutase,
Glyma.02G095500	Glyma4.0	Sulfate transporter 4.1
Glyma.05G038100	Glyma4.0	Iron-sulfur cluster assembly protein
Glyma.16G030800	Glyma4.0	S-adenosyl-L-methionine-dependent methyltransferases superfamily protein
Glyma.02G095500	Glyma4.0	Sulfate transporter 4.1
Glyma.04G080300	Glyma4.0	Sulfite exporter TauE/SafE family protein

4. Discussion

4.1 QTL Analysis for N, P, and S

The significant effects of line and location is due to the genotypic differences among lines and the contribution of the environment to the variation of the trait. The high heritability of 91.7% of seed N trait variation is due to genetics, followed by 48.2% for S seed concentration trait, and finally an inheritance of close to zero for seed P concentration trait. The very low inheritance, recorded in seed P may indicate the strong effect of environmental factors on the trait and very little effects due to genetic differences. The low heritability that was shown in seed P could be due to complex quantitative trait, gene-to-gene interactions (epistasis effect), and significant gene by environment interactions with P, and suggest limitations of these QTL use in the breeding selection [28]. It was reported on other minerals that the non-genetic factors could be extremely high [28, 29]. They further added that the low heritability could be the trait quantitatively inherited, multiple gene with small effects significantly interact with the environment to produce the phenotype. Also, the large interaction of gene with environment for P QTL may require a large number of RILs across locations and years to obtain significant QTL with high inheritance [28,30]. The positive correlations between nutrients in both years and at both locations indicated the symport relationship between these nutrient transport system. The negative correlation between N and P in 2018 indicated antiport relationship that can occur due to environmental factors, especially drought, heat, and soil conditions. Positive and negative mineral relations were previously observed ~~previously~~ [11,12,43,44,45].

To our knowledge p~~P~~revious genomic research, conducted on minerals, was mostly conducted ~~research reported that most of the genomic research and genetic mapping, that are conducted on minerals, were conducted~~ on leaves, roots, or shoot [3,15,16], and not on seed nutrients accumulation in soybean seeds [3,17,18]. For example, searching SoyBase (https://www.soybase.org/search/index.php?searchterm=Nitrogen+and+Phosphorus+and+Sulfur&list=bi_parental_qtl_listview) revealed that only QTL and molecular markers related to N, P, and S concentrations in shoot tissue were identified [17]. They were able, using GWAS and 31,748 SNPs, to identify soybean shoot QTL, including one QTL for P (*qPHO001*), one QTL for N (*qNIT001*), one QTL for S (*qSUL001*). They explained that QTL clustering of P, K, Mg, C, N, and S indicated physiological and genetic relationships, and possible similar metabolic processes between these nutrients. Other researchers identified QTL related to leaf minerals and were able to identify, using 200 RILs, 6366 SNPs markers that covered the whole genome, and 19 QTL and 3 candidate genes associated to leaf-related traits [19]. Previous research, using 92 F5:7 recombinant inbred lines, a cross between MD 96-5722 (MD) and Spencer, and 5,376 SNP markers, were also able to detect QTL related to seed N, P, and S [23]. They identified N QTL *qNIT001* on Chr 16 at the marker interval ss248983974-ss248977568 with ~~, and~~ LOD of 9.85 and a contribution to phenotypic variance of 94%~~, with a positive additive effect of 3.25~~ [23]. They were also identified P QTL *qPHO001* on Chr16 at marker interval ss248983974-ss248977568~~,~~ with LOD of 9.49 and a contribution to phenotypic variance of 94%~~' and a positive additive effect of 0.25~~. The S QTL *q SUL001* on Chr16 was also detected at the marker interval ss248983974-ss248977568 with a LOD peak of 9.73 and a contribution to the phenotypic variation of 94% [23]. ~~and positive additive effects of 0.15.~~ G~~Also, g~~enomic regions associated with seed N accumulation in soybean at R5, R6, and R7 growth stages were also

identified [19]. They used a population of 101 F6:8 RILs, a cross between N87-984-16 x TN93-99 [19]. They detected QTL on Chr 2, 7, 8, 14, 15, 18, and the contribution to the phenotypic variation ranged from 5 to 11.6%. Other researchers worked on genomic regions for other mineral such as P. For example, using 184 RILs, a cross between Kefeng No. 1 and Nanong 1138-2 soybean varieties, others [16] were able to identify QTL for P deficiency tolerance in leaves, roots, and shoots, and identified 7 QTL associated with weight of fresh shoot, phosphorus contents in leaf and in root. Other also identified P QTL associated with shoot P accumulation [20,21,22]. Our research showed that, except for QTL detected on Chr 16 [23], 11 QTL reported here were not previously identified, therefore, they are novel.

4.2 Candidate Genes and Gene Annotation for N P S

Candidate genes were identified for N, P, and S concentrations in soybean seeds were annotated using SoyBase Genome Browser (glyma.Wm82.gnm4) (**Table 4 and Table 5**). These annotated genes were located either within the identified QTL interval or vicinity areas. For N, more than 11 genes, including ammonium transporter, nodulin like proteins, glutamine-dependent NAD (+) synthetase, ammonium transmembrane transporters were identified in the functional loci, suggesting the functional association with N metabolism and accumulation and their genetic variants. These candidate genes may play roles in ammonium transport across cellular membranes, reversible transfer of an amino group between alanine and α-ketoglutarate and forming glutamate and root nodules of leguminous plants. The ammonium transporters (AMTs) are important genes involved in ammonium absorption and utilization in soybeans. The QTL underlying N concentration were identified on chromosome 2 and 10 in this research and

explained more than 8 % and 42% phenotypic variation, respectively. The candidate genes associated with these two loci, *Glyma.02G043700* and *Glyma.10G030800* were identified in the intervals. The same candidate genes *GmAMT4.4* (*Glyma.02G043700*) and *GmAMT4.6* (*Glyma.10G030800*) were also annotated, and genes expression of *GmAMT* family genes in the soybean plants, using a GWAS, was conducted by others [46]. Both candidate genes were among 16 *GmAM* paralog genes annotated in the study and GmAMT4.6 was confirmed to be related with the circadian rhythms in the transcription analysis [46]. Moreover, there were several candidate genes existing within the loci for the traits of P and S, suggesting the functional significance of these intervals for the nutrients in soybean. The candidate gene inositol-tetrakisphosphate 1-kinase 2-like isoform X1 (*Glyma.04G088000*) was annotated in Soybase involved in the metabolism of inositol phosphates as the signaling molecules. Protein phosphatase 2C family gene (*Glyma.03G043675*) appeared to be involved in the regulation of crucial steps in the cells by catalyzing the removal of phosphate groups from proteins. The candidate gene purple acid phosphatase 17 (*Glyma.20G011900*) under the QTL (*qP-03*) on chromosome 20 is involved in phosphate metabolism. The QTL (*qP-03*) explained more than 5% phenotypic variation. Zhu et al. (2020)[47] studied the dynamic changes of intracellular (leaf and root) and root-associated APase (purple acid phosphatase) activity under both Pi-sufficient and Pi-deficient conditions for a total of 38 purple acid phosphatase (*GmPAP*) members identified in the soybean genome. The relative expression levels of *GmPAP17c* (*Glyma.20G011900*) and other *GmPAP*s in the leaves and roots were analyzed at two P levels using qRT-PCR analysis. However, the transcript abundance of the *GmPAP17c* was detected at both P levels in the leaves, but not in the roots[47]. There were several candidate genes identified for the sulfur content within the QTL intervals. The sulfate transporter 4.1 (*Glyma.02G095500*) underneath (*qS-01*) is

involved in the transport of sulfate ions across cell membranes. The iron-sulfur cluster assembly protein (Gm05_3273418) is involved in electron transfer reactions and other redox processes.

It is clear, based on the above discussion, and except of seed N, P, and S QTL, detected on Chr 16 [23], no QTL of seed N, P, and S were previously detected in mature seed for the accumulation of these nutrients. Therefore, our research showed that 11 QTL reported here were not previously identified, therefore, they are novel.

5. Conclusions

In this research a total of 12 QTL were identified for seed N, P, and S at the complete physiological seed maturity (R8), and among which, 11 QTL were novel. Seed N, P, and S on Chr 16 were previously identified by others. QTL and molecular markers discovered in this research will help breeders for selection for optimum mineral nutritional qualities; for physiologists to advance our knowledge in the physiology and genetics of seed mineral accumulation, and for molecular biologists with new knowledge on seed N, P, and S candidate genes and their possible use in genetic engineering.

Supplementary Materials: Figure (S1-S16) and their titles are presented and available.

Author Contributions: Conceptualization, M.A.K. and K.M.; methodology, D.K., J.Y., T.V., M.U., Q.S., F.B., T.R., E.W., N.L., N.B. and A.M.; validation, M.A.K., K.M. and H.T.N.; formal analysis, D.K., J.Y. A.M., and N.B.; investigation, K.M., D.K. and A.M.; resources, K.M., N.B.,

A.M., and H.T.N.; genotyping, Q.S., data curation, D.K., J.Y., M.U. and T.V.; writing—original draft preparation, N.B., M.A.K. and D.K.; writing—review and editing, D.K., J.Y., N.B., N.L., H.M., T.V., A.M., M.A.K., K.M. and H.T.N.; visualization, J.Y.; supervision, M.A.K., J.Y., K.M., H.M., H.T.N.; project administration, M.A.K., K.M., H.T.N., N.B. All authors have read and agreed to the published version of the manuscript.

Funding: This research was funded by the U.S. Department of Agriculture, Agricultural Research Service Project 6066-21220-016-000D, SIUC, UM, and FSU. All authors have read and agreed to the published version of the manuscript.

Data Availability Statement: Data supporting reported results are available on request from the corresponding author.

Acknowledgments: We would like to thank Tri D. Vuong for his technical support on the SoySNP6K Infinium genotyping and linkage map construction. Technical support provided by Sandra Mosley and Rama Gadi is also appreciated. Mention of trade names or commercial products in this publication is solely for the purpose of providing specific information and does not imply recommendation or endorsement by the United States Department of Agriculture (USDA). The findings and conclusions in this publication are those of the authors and should not be construed to represent any official USDA or U.S. Government determination or policy. USDA is an equal opportunity provider and employer.

Conflicts of Interest: The authors declare no conflict of interest.

References

1. Chu, Q, Watanabe T, Shinano T, Nakamura T, Oka T, Osaki M, Sha Z. The dynamic state of the ionome in roots, nodules, and shoots of soybean under different nitrogen status and at different growth stages. *J. Plant Nutr. Soil Sci.* **2016**, *179*, 488–498.
2. Ray JD, Fritschi FB, Soybean mineral nutrition and biotic relationships. In: *Fertilizers: Properties, applications and effects.* Elsworth L.R., Paley W.O., Eds.; Nova Science Publishers Inc., New York, **2009**; p. 29–56.
3. Dhanapal AP, Ray JD, Smith JR, Purcell LC, Fritschi FB, Identification of novel genomic loci associated with soybean shoot tissue macro and micronutrient concentrations. *Plant Genome*. **2018**, *11*, 170066.
4. Shibles RM, Sundberg DN, Naeve SL. Physiological constraints limiting seed protein in normal and high seed protein soybean genotypes. *Agron. Abstr.* **1994**, 151.
5. Staswick PE, Zhang Z, Clemente TE, Specht JE. Efficient down-regulation of the major vegetative storage protein genes in transgenic soybean does not compromise plant productivity. *Plant Physiol.* **2001**, *127*, 1819–1826.
6. Imsande J. Selection of soybean mutants with increased concentrations of seed methionine and cysteine. *Crop Sci.* **2001**, *41*, 510–515.
7. Gayler KR, Sykes GE. β-Conglycinins in developing soybean seeds. *Plant Physiol.* **1981**, *67*, 958–961.

8. Kumudini S, Hume DJ, Chu G. Genetic improvement in short-season soybeans: II. Nitrogen accumulation, remobilization, and partitioning, *Crop Sci.* **2002**, *42*, 141–145.
9. Nakasathien S, Israel DW, Wilson RF, Kwanyuen P. Regulation of seed protein concentration in soybean by supra-optimal nitrogen supply, *Crop Sci.* **2000**, *40*, 1277–1284.
10. Barker AV, Pilbeam DJ. *Handbook of Plant Nutrition*, Boca Raton, FL, CRC Press, **2007**; pp. 19-199.
11. Marschner H. Mineral nutrition of higher plants. *J. Ecol*. **1995**, *76*, 1250.
12. Marschner P. Marschner's Mineral Nutrition of Higher Plants. **2012.** Third Edition, Elsevier Ltd.
13. Wang X, Jiang G, Song Q, Cregan P, Scott R, Zhang J, Yen Y, Brown M. Quantitative trait locus analysis of seed sulfur-containing amino acids in two recombinant inbred line populations of soybean. *Euphytica*, **2015**, *201*, 293-305.
14. Soybase: https://www.soybase.org/search/index.php?searchterm=Nitrogen+and+Phosphorus+and+Sulfur&list=bi_parental_qtl_listview
15. Liu DL, Chen SW, Liu XC, Yang F, Liu WG, She YH, Du JB, Liu CY, Yang WY, Wu XL. Genetic map construction and QTL analysis of leaf-related traits in soybean under monoculture and relay intercropping. *Sci. Rep.* **2019**, *9*, 2716.
16. Li YD, Wang YJ, Tong YP, Gao JG, Zhang JS, Chen SY. QTL mapping of phosphorus deficiency tolerance in soybean (*Glycine max* L. Merr.) *Euphytica*, **2005**, *142*, 137-142.
17. King KE, Peiffer GA, Reddy M, Lauter N, Lin SF, Cianzio S, Shoemaker RC. Mapping of iron and zinc quantitative trait loci in soybean for association to iron deficiency

chlorosis resistance. *J. Plant Nutr*. **2013**, *36*, 2132–2153. DOI: 10.1080/01904167.2013.766804

18. Ramamurthy RK, Jedlicka J, Graef GL, Waters BM. Identification of new QTLs for seed mineral, cysteine, and methionine concentrations in soybean [*Glycine max* (L.) Merr.]. *Mol. Breed*. **2014**, *34*, 431–445.
19. Panthee DR, Pantalone VR, Sams CE, Saxton AM, West DR, Rayford WE. Genomic regions governing soybean seed nitrogen accumulation. *J. Am. Oil. Chem. Soc.* **2004**, *81*, no. 1
20. Zhang D, Li H, Wang J, Zhang H, Hu Z, Chu S, Lv H, Yu D. High-density genetic mapping identifies new major loci for tolerance to low-phosphorus stress in soybean. *Front. Plant Sci*. **2016**, *7*, 372. https://doi.org/10.3389/fpls.2016.00372
21. Liang Q, Cheng X, Mei M, Yan X, Liao H. QTL analysis of root traits as related to phosphorus efficiency in soybean. *Ann. Bot*. **2010**, *106*, 223-234.
22. Walker DR, Scaboo AM, Pantalone VR, Wilcox JR, Boerma HR. Genetic mapping of loci associated with seed phytic acid content in CX1834-1-2 Soybean. *Crop Sci*. **2006**, *46*, 390-397.
23. Bellaloui N, Khandaker L, Akond M, Kantartzi SK, Meksem K, Mengistu M, Lightfoot, DA, Kassem MA. Genetic mapping of QTL associated with seed macronutrients accumulation in 'MD 96-5722' by 'Spencer' recombinant inbred lines of soybean. *Atlas J. Biol*. **2015,** *3*, 224–235.
23. ~~Identification of QTL underlying seed micronutrients accumulation in 'MD 96-5722' by 'Spencer' recombinant inbred lines of soybean. *Atlas J. Plant Biol.* **2015**, 39–49, doi: 10.5147/ajpb.2015.0155~~

Formatted: Font: Italic

Formatted: Font: Bold

Formatted: Font: Italic

24. Shultz JL, Kurunam D, Shopinski K, Iqbal MJ, Kazi S, Zobrist K, Bashir R, Yaegashi S, Lavu N, Afzal AJ, et al. The soybean genome database (SoyGD): A browser for display of duplicated, polyploid, regions and sequence tagged sites on the integrated physical and genetic maps of Glycine max. *Nucleic Acids Res*. **2006**, *34*, D758–D765.

25. Hartwig EE, Epps JM. Registration of 'Forrest' soybeans. *Crop. Sci.* **1973**, *13*, 287.

26. Bernard, R.L.; Cremeens, C.R. Registration of Williams 82 soybean. *Crop. Sci*. **1988**, *28*, 1027–1028.

27. Knizia D, Yuan J, Bellaloui N, Vuong T, Usovsky M, Song Q, Betts F, Register T, Williams E, Lakhssassi N, Mazouz H, Nguyen HT, Meksem K, Mengistu A, Kassem MA. The soybean high density 'Forrest' by 'Williams 82' SNP-based genetic linkage map identifies QTL and candidate genes for seed isoflavone content. *Plants* **2021**, *10*, 2029.

28. Bellaloui N, Knizia D, Yuan J, Song Q, Betts F, Register T, Williams E, Lakhssassi N, Mazouz H, Nguyen HT, Meksem K, Mengistu A, Kassem MA. Genetic mapping for QTL associated with seed nickel and molybdenum accumulation in the soybean 'Forrest' by 'Williams 82' RIL Population. *Plants* **2023**, *12*, 3709. https://doi.org/10.3390/plants12213709

29. Kassem, M.A. Seed amino acids, macronutrients, micronutrients, sugars, and other compounds. In Soybean Seed Composition: Protein, Oil, Fatty Acids, Amino Acids, Sugars, Mineral Nutrients, Tocopherols, and Isoflavones; Springer: Cham, Switzerland, **2021**. ~~Kassem~~

Formatted: Font: Bold

30. Bellaloui N, Knizia D, Yuan J, Song Q, Betts F, Register T, Williams E, Lakhssassi N, Mazouz H, Nguyen HT, Meksem K, Mengistu A, Kassem MA. Genomic regions and

candidate genes for seed iron and seed zinc accumulation identified in the soybean 'Forrest' by 'Williams 82' RIL Population. *Int. J. Plant Biol*. **2024**, *15*, 452-467. https://doi.org/10.3390/ijpb15020035

31. Bellaloui N, Smith JR, Gillen AM, Ray JD. Effects of maturity, genotypic background, and temperature on seed mineral composition in near-isogenic soybean lines in the early soybean production system. *Crop Sci*. **2011**, *51*, 1161–1171.
32. Bellaloui N, Turley RB. Effects of fuzzless cottonseed phenotype on cottonseed nutrient composition in near isogenic cotton (*Gossypium hirsutum* L.) mutant lines under well-watered and water stress conditions. *Front. Plant Sci*. **2013**, *4*, 1-13.
33. Cavell AJ. The colorimetric determination of phosphorus in plant materials. *J. Sci. Food Agric*. **1955**, *6*, 479–480.
34. Vuong TD, Sleper DA, Shannon JG, Nguyen HT. Novel quantitative trait loci for broad-based resistance to soybean cyst nematode (*Heterodera glycines* Ichinohe) in soybean PI 567516C. *Theor. Appl. Genet*. **2010**, *121*, 1253–1266.
35. Song Q, Yan L, Quigley C, Fickus E, Wei H, Chen L, Dong F, Araya S, Liu J, Hyten D, Pantalone V, Nelson RL. Soybean BARCSoySNP6K: An assay for soybean genetics and breeding research. *Plant J*. **2020**,*104*, 800-811.
36. Grant D, Nelson RT, Cannon SB, Shoemaker RC. SoyBase, the USDA-ARS soybean genetics and genomics database. *Nucleic Acids Res*. **2010**, *38*, D843–D846.
37. Brown AV, Conners SI, Huang W, Wilkey AP, Grant D, Weeks NT, Cannon SB, Graham MA, Nelson RT. A new decade and new data at SoyBase, the USDA-ARS soybean genetics and genomics database. *Nucleic Acids Res*. **2020**, *49*, D1496–D1501.

38. Pilet-Nayel ML, Muehlbauer FJ, McGee RJ, Kraft JM, Baranger A, Coyne CJ. Quantitative trait loci for partial resistance to Aphanomyces root rot in pea. *Theor. Appl. Genet.* **2002**, *106*, 28–39.

39. R Software. Version R 4.2.0. Available online: https://www.r-project.org (accessed on 15 June 2023).

40. Wang S, Basten CJ, Zeng ZB. *Windows QTL Cartographer 2.5*; Department of Statistics, NCSU: Raleigh, NC, USA, 2012; Available online: http://statgen.ncsu.edu/qtlcart/WQTLCart.htm (accessed on 8 August 2023).

41. Voorrips RE. MapChart: Software for the graphical presentation of linkage maps and QTL. *J. Hered.* **2002**, *93*, 77–78.

42. SAS Institute. *Statistical Analysis Systems (SAS)*; SAS Institute: Cary, NC, USA, **2002–2012**.

43. Howell RW, Cartter JL. Physiological factors affecting composition of soybeans: I. Correlation of temperature during certain portions of the pod filling stage with oil percentagein mature beans. *Agron. J*. **1953**, *45*, 526‐528.

44. Engels C, Munkle L, Marschner H. 1992. Effect of root zone temperature and shoot demand on uptake and xylem transport of macronutrients in maize (*Zea mays* L.). J. *Exp.Bot*. 44:1695‐1701.

45. Gent MPN, Ma YZ. Mineral nutrition of tomato under diurnal temperature variation of root and shoot. *Crop Sci.* **2000**, *40*, 1629‐1636.

46. Yang W, Dong X, Yuan Z, Zhang Y, Li X, Wang Y. Genome-wide identification and expression analysis of the ammonium transporter family genes in soybean. *Int. J. Mol. Sci*. **2023**, *24*, 3991, doi: 10.3390/ijms24043991.

47. Zhu S, Chen M, Liang C, Xue Y, Lin S, Tian J. Characterization of purple acid phosphatase family and functional analysis of GmPAP7a/7b involved in extracellular ATP utilization in soybean. *Front. Plant Sci*. **2020**, *11*, 1-15, doi: 10.3389/fpls.2020.00661.

 Click here to access/download;Figure;Figure 1.TIF

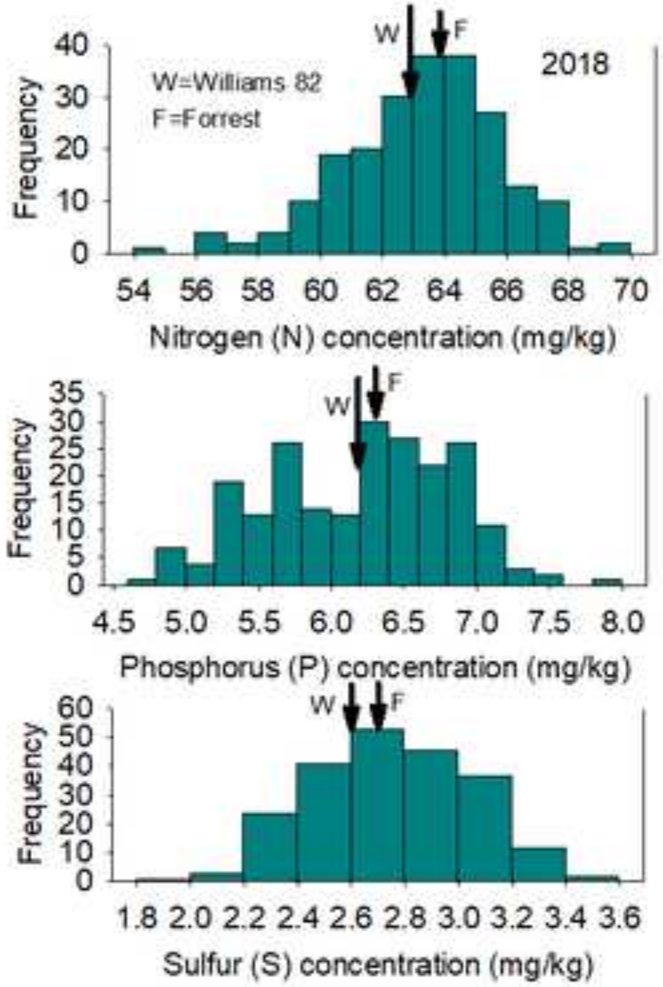

Click here to access/download;Figure;Figure 2.TIF

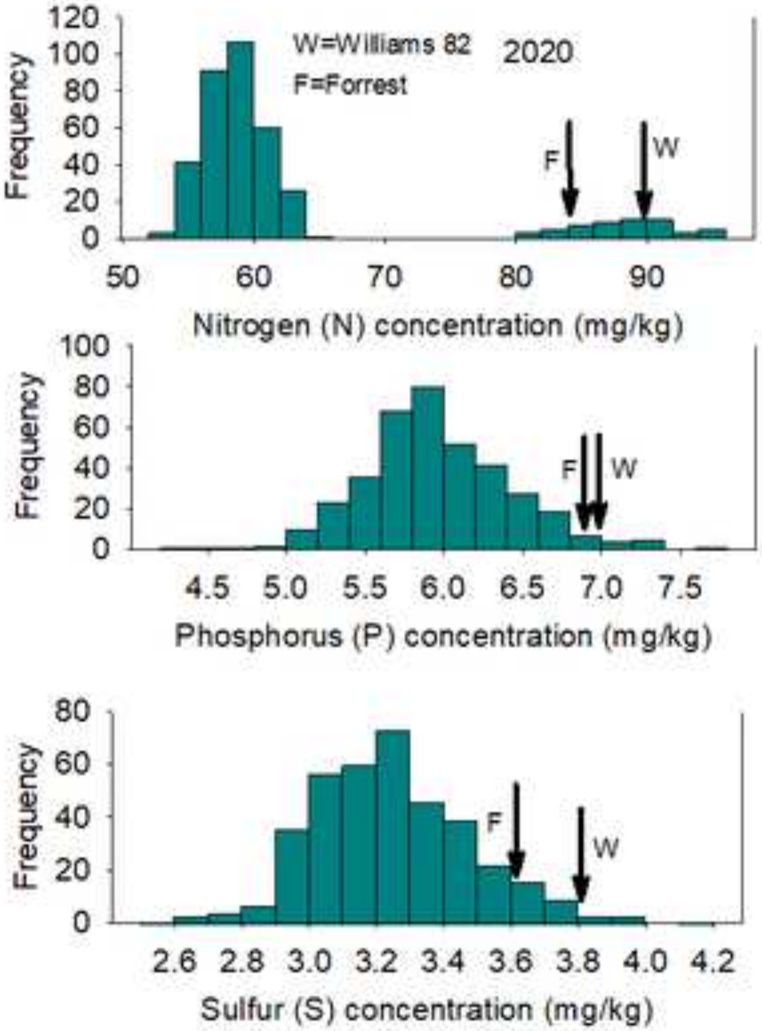

Figure
Click here to access/download;Figure;Figure 3.TIF

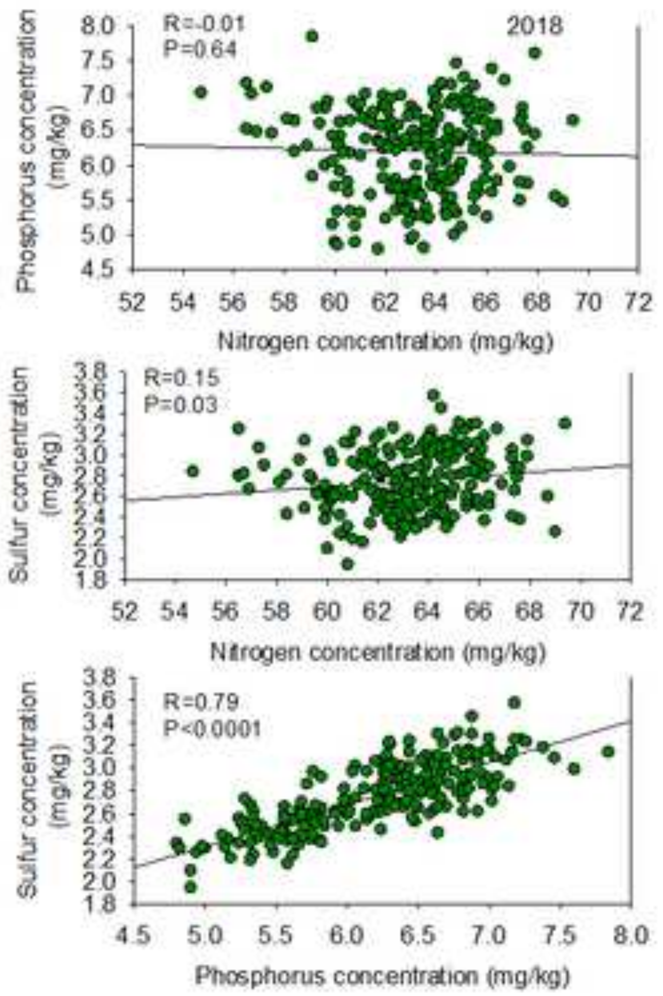

 Click here to access/download;Figure;Figure 4.TIF

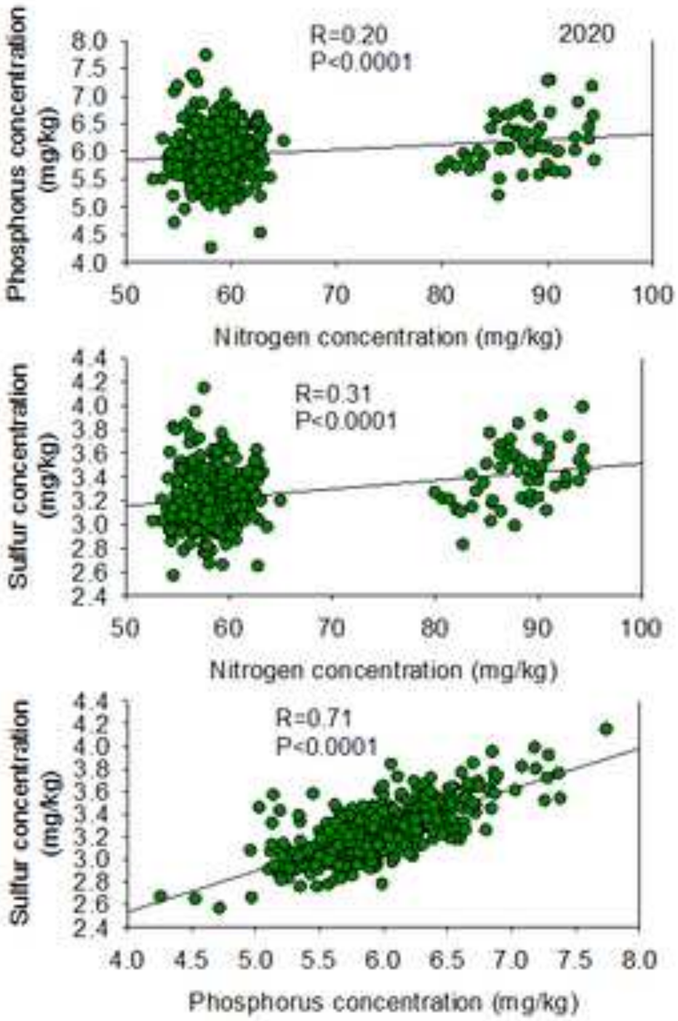

Supplementary Figures

Click here to access/download
Supporting Information
Supplimentary Material-Figures.docx
