# Supplementary material for: Genomic regions and candidate genes associated with seed nitrogen, phosphorus, and sulfur accumulation identified in the soybean ‘Forrest’ by ‘Williams 82’ RIL population
Source: PLoS One. 2025 Sep 3;20(9):e0331214. doi: 10.1371/journal.pone.0331214 (PMC12407463; doi:10.1371/journal.pone.0331214)
Supplement: Attachment — Submitted filename: Response to Reviewers.docx [file pone.0331214.s021.docx]

Dear Dr. Hao-Xun Chang, Academic Editor, PLOS ONE:

Please receive our revised manuscript entitled “Genomic Regions and Candidate Genes Associated with Seed Nitrogen, Phosphorus, and Sulfur Accumulation Identified in the Soybean ‘Forrest’ by ‘Williams 82’ RIL Population” by Bellaloui et al.

We revised the manuscript and addressed all the reviewers’ comments as instructed, on one-by-one basis, and all are track-changes. We also addressed the comments noted in the attached file (form Reviewer 1) either as suggested or as appropriate. Our responses are below. We stated the reviewer’s comments and responded to them, as shown below. Enormous efforts and time were devoted in revising this manuscript, and we believe the reviewers’ comments improved the quality of the manuscript. We would like to note that “ The funders had no role in study design, data collection and analysis, decision to publish, or preparation of the manuscript."

Thank you and we appreciate your service,

**Reviewers comments and Authors’ Responses**

Journal Requirements:

All journal requirements were addressed.

1. When submitting your revision, we need you to address these additional requirements.

Please ensure that your manuscript meets PLOS ONE's style requirements, including those for file naming. The PLOS ONE style templates can be found at

[https://journals.plos.org/plosone/s/file?id=wjVg/PLOSOne_formatting_sample_main_body.pdf](https://gcc02.safelinks.protection.outlook.com/?url=https%3A%2F%2Fjournals.plos.org%2Fplosone%2Fs%2Ffile%3Fid%3DwjVg%2FPLOSOne_formatting_sample_main_body.pdf&data=05%7C02%7Cnacer.bellaloui%40usda.gov%7C7b8579dbdef148c7664608dda33ebebf%7Ced5b36e701ee4ebc867ee03cfa0d4697%7C1%7C0%7C638846212451530075%7CUnknown%7CTWFpbGZsb3d8eyJFbXB0eU1hcGkiOnRydWUsIlYiOiIwLjAuMDAwMCIsIlAiOiJXaW4zMiIsIkFOIjoiTWFpbCIsIldUIjoyfQ%3D%3D%7C0%7C%7C%7C&sdata=nv1yOBTU%2Bsk1Bd18%2BcQMYfA2M%2BFmFx4ps9Sf8kjiSV4%3D&reserved=0) and
[https://journals.plos.org/plosone/s/file?id=ba62/PLOSOne_formatting_sample_title_authors_affiliations.pdf](https://gcc02.safelinks.protection.outlook.com/?url=https%3A%2F%2Ftrack.editorialmanager.com%2FCL0%2Fhttps%3A%252F%252Fjournals.plos.org%252Fplosone%252Fs%252Ffile%253Fid%3Dba62%252FPLOSOne_formatting_sample_title_authors_affiliations.pdf%2F1%2F010f019656dd8154-8314bb73-4e99-4fcc-9087-3ad96b0d3170-000000%2FK_CEHM8qh2nafgCTn0euehQIQ9Lq_z3eCddWRwL9mg4%3D207&data=05%7C02%7Cnacer.bellaloui%40usda.gov%7C7b8579dbdef148c7664608dda33ebebf%7Ced5b36e701ee4ebc867ee03cfa0d4697%7C1%7C0%7C638846212451543190%7CUnknown%7CTWFpbGZsb3d8eyJFbXB0eU1hcGkiOnRydWUsIlYiOiIwLjAuMDAwMCIsIlAiOiJXaW4zMiIsIkFOIjoiTWFpbCIsIldUIjoyfQ%3D%3D%7C0%7C%7C%7C&sdata=L4kv0djc4qIYagxOoLDmLBA%2F4N3uscBNoP0yjSBCSkg%3D&reserved=0)

Authors’ response

We revised the entire manuscript, and fixed the files’ names for tables, fugues, files, and so on according to the journal requirements.

2. We noticed you have some minor occurrence of overlapping text with the following previous publication(s), which needs to be addressed:

[https://www.mdpi.com/2037-0164/15/2/35](https://gcc02.safelinks.protection.outlook.com/?url=https%3A%2F%2Ftrack.editorialmanager.com%2FCL0%2Fhttps%3A%252F%252Fwww.mdpi.com%252F2037-0164%252F15%252F2%252F35%2F1%2F010f019656dd8154-8314bb73-4e99-4fcc-9087-3ad96b0d3170-000000%2F-uOwKL8RIKG7qx1HoPgd9-1hvRauFrSWV6Fs2pSQCwY%3D207&data=05%7C02%7Cnacer.bellaloui%40usda.gov%7C7b8579dbdef148c7664608dda33ebebf%7Ced5b36e701ee4ebc867ee03cfa0d4697%7C1%7C0%7C638846212451558016%7CUnknown%7CTWFpbGZsb3d8eyJFbXB0eU1hcGkiOnRydWUsIlYiOiIwLjAuMDAwMCIsIlAiOiJXaW4zMiIsIkFOIjoiTWFpbCIsIldUIjoyfQ%3D%3D%7C0%7C%7C%7C&sdata=Mb2WQnWxJotCrPYFHXnhYhpUU7kN6pS%2BTT7VYgjfUAc%3D&reserved=0)

In your revision ensure you cite all your sources (including your own works), and quote or rephrase any duplicated text outside the methods section. Further consideration is dependent on these concerns being addressed.

Authors’ response

We revied the manuscript and made sure there are no similarities, unless the similarities are scientific terms or technical names. In addition, that published manuscript dealt with Fe and Zn, but the current manuscript deals with N, S, and P.

3. Thank you for stating the following financial disclosure:

 [This research was funded by the U.S. Department of Agriculture, Agricultural Research Service Project 6066-21220-016-000D, SIUC, UM, and FSU.].

Please state what role the funders took in the study.  If the funders had no role, please state: ""The funders had no role in study design, data collection and analysis, decision to publish, or preparation of the manuscript."

We include the appropriate statement in the manuscript as instructed “

Authors’ response

“The funders had no role in study design, data collection and analysis, decision to publish, or preparation of the manuscript."

If this statement is not correct you must amend it as needed.

Please include this amended Role of Funder statement in your cover letter; we will change the online submission form on your behalf.

Authors’ response

We included it in the cover letter as well, as below:

“ The funders had no role in study design, data collection and analysis, decision to publish, or preparation of the manuscript."

4. When completing the data availability statement of the submission form, you indicated that you will make your data available on acceptance. We strongly recommend all authors decide on a data sharing plan before acceptance, as the process can be lengthy and hold up publication timelines. Please note that, though access restrictions are acceptable now, your entire data will need to be made freely accessible if your manuscript is accepted for publication. This policy applies to all data except where public deposition would breach compliance with the protocol approved by your research ethics board. If you are unable to adhere to our open data policy, please kindly revise your statement to explain your reasoning and we will seek the editor's input on an exemption. Please be assured that, once you have provided your new statement, the assessment of your exemption will not hold up the peer review process.

N/A

Reviewers' comments:

Reviewer's Responses to Questions

**Comments to the Author**
1. Is the manuscript technically sound, and do the data support the conclusions?
The manuscript must describe a technically sound piece of scientific research with data that supports the conclusions. Experiments must have been conducted rigorously, with appropriate controls, replication, and sample sizes. The conclusions must be drawn appropriately based on the data presented.

Reviewer #1: Yes

Reviewer #2: Yes

Authors’ response

Thank you for the comment.

2. Has the statistical analysis been performed appropriately and rigorously?

Reviewer #1: Yes

Reviewer #2: Yes

Authors’ response

Thank you for the comment.

3. Have the authors made all data underlying the findings in their manuscript fully available?

The [PLOS Data policy](https://gcc02.safelinks.protection.outlook.com/?url=http%3A%2F%2Ftrack.editorialmanager.com%2FCL0%2Fhttp%3A%252F%252Fwww.plosone.org%252Fstatic%252Fpolicies.action%2523sharing%2F1%2F010f019656dd8154-8314bb73-4e99-4fcc-9087-3ad96b0d3170-000000%2FmMei7cbWyTt95u6aOo8ACFtWyIZcvTJB7UdKs1qxXDE%3D207&data=05%7C02%7Cnacer.bellaloui%40usda.gov%7C7b8579dbdef148c7664608dda33ebebf%7Ced5b36e701ee4ebc867ee03cfa0d4697%7C1%7C0%7C638846212451571477%7CUnknown%7CTWFpbGZsb3d8eyJFbXB0eU1hcGkiOnRydWUsIlYiOiIwLjAuMDAwMCIsIlAiOiJXaW4zMiIsIkFOIjoiTWFpbCIsIldUIjoyfQ%3D%3D%7C0%7C%7C%7C&sdata=kr0MzMJNt1zNjXubwkaF7Y4ig7KeFjvf%2FXUwG6gd9mU%3D&reserved=0) requires authors to make all data underlying the findings described in their manuscript fully available without restriction, with rare exception (please refer to the Data Availability Statement in the manuscript PDF file). The data should be provided as part of the manuscript or its supporting information, or deposited to a public repository. For example, in addition to summary statistics, the data points behind means, medians and variance measures should be available. If there are restrictions on publicly sharing data—e.g. participant privacy or use of data from a third party—those must be specified.

Reviewer #1: Yes

Reviewer #2: Yes

Authors’ response

Thank you for the comment.

4. Is the manuscript presented in an intelligible fashion and written in standard English?

PLOS ONE does not copyedit accepted manuscripts, so the language in submitted articles must be clear, correct, and unambiguous. Any typographical or grammatical errors should be corrected at revision, so please note any specific errors here.

Reviewer #1: Yes

Reviewer #2: No

Authors’ response

We revised the manuscript and made sure the presentation and English writing is sound.

5. Review Comments to the Author

Please use the space provided to explain your answers to the questions above. You may also include additional comments for the author, including concerns about dual publication, research ethics, or publication ethics. (Please upload your review as an attachment if it exceeds 20,000 characters)

Reviewer #1: The trait seed P content has near zero heritability meaning no genotypic variation for the trait. Then all the analysis will be wrong. How to justify your study on this trait. There is some English corrections in the manuscript which needs to attended. There is markings done in the manuscript which needs to be attended. Some discussion is excessively written which needs to addressed before the final acceptance of the research paper.

Our response

We appreciate the reviewer’s important observation regarding the low broad-sense heritability (H² ≈ 0) for seed phosphorus (P) content. Indeed, low heritability suggests that the phenotypic variation is primarily governed by environmental effects and/or strong genotype-by-environment (G×E) interactions, which complicates genetic dissection. However, we respectfully argue that this does not invalidate the analysis, for the several below reasons (we further explained the below concepts in the Discussion section for the benefit for the reader, and all are track-changed).

1. **Biological and Agricultural Relevance**
   Seed P content remains a biologically and nutritionally important trait in soybean, especially due to its connection with seed vigor, germination, and nutritional quality. Even when heritability is low, understanding the genetic basis of this trait is critical for identifying genotypes or genomic regions responsive to environmental P variability.
2. **Precedent in Literature**
   Low heritability for P-related traits in soybean has been reported previously, especially under single-environment studies or limited replication. For instance, Li et al. (2005) and Zhang et al. (2016) both reported variable P heritability depending on tissue type, environment, and developmental stage. These studies still identified meaningful QTL by using multi-location or repeated trials; similar to our approach.
3. **Detection of Stable QTL**
   Despite low overall heritability, we were able to identify several statistically significant QTL for seed P content in both environments (see Table 3), with LOD values ranging from 2.5 to 9.4 and phenotypic variance explained (R²) up to 12.17%. This suggests that specific loci still contribute consistently across environments, even if the overall trait expression is environmentally labile.
4. **Justification Through G×E**
   The low heritability itself is an important genetic insight. It indicates a potential for strong environmental modulation or G×E interactions, which could be exploited through genotype-specific agronomic strategies or P management practices. Moreover, inclusion of this trait helps distinguish which QTL are environment-stable versus those that are more plastic.

References used above are below, and they are included in the manuscript.

Li, Y.D., Wang, Y.J., Tong, Y.P., Gao, J.G., Zhang, J.S., & Chen, S.Y. (2005). QTL mapping of phosphorus deficiency tolerance in soybean (Glycine max L. Merr.). Euphytica, **142**, 137–142. <https://doi.org/10.1007/s10681-005-1047-2>.

Zhang, D., Li, H., Wang, J., Zhang, H., Hu, Z., Chu, S., Lv, H., & Yu, D. (2016). High-density genetic mapping identifies new major loci for tolerance to low-phosphorus stress in soybean. Frontiers in Plant Science, **7**, 372. <https://doi.org/10.3389/fpls.2016.00372>.

Reviewer #2: The manuscript aims to identify new genomic regions associated with seed nitrogen, phosphorus, and sulfur accumulation with the Forrest by Williams 82 RIL mapping population. Several references were cited in the manuscript without the background information in the materials and methods, it would be beneficial if more in-depth information was provided throughout the article. While the manuscript is insightful, it requires major revision before it can be published. Some examples are listed below.

Authors response:

We revised the entire manuscript for better comprehension and further in-depth information, and we paid attention to the cited references in Materials and methods for further details and backgrounds. All edits are track-changes.

Introduction:
A good start for the introduction, more background information should be provided. For example, the introduction should briefly discuss the articles referenced, it would be best to directly reference the authors (Line 81 for example, which author; similarly in Line 97, directly citing the author would make the article easier to follow along) Several editorial recommendations include:

Authors response:

The introduction was revised and adjusted as needed for better clarification. Citations were directly added for better clarification. We worry that adding more information will be in conflict with the suggestion of Reviewer 1 as Reviewer 1 indicated that some discussion is excessively written.

Line 66 Soybean missing scientific name
Line 85 full name of the essential nutrients
Line 88 missing , in 6366
Line 97 F6:8
Line 98 N87-984-16 x TN93-99 => × (cross)
Line 98 Chrs 2, 7, 8, 14, 15, 18, suggest including linkage group after the chromosome numbers
Line 99 others (who?)
QTL first appears in article -> Quantitative trait loci
Line 101 “seven” QTL
Line 102 phosphorus => P
Line 103 F5:7

Authors response:

The above suggested comments were addressed as suggested. We included the linkage group in all tables.

Materials and Methods
Reviewer’s comment

Are there any reasons why this mapping population was selected for the study? Forrest was initially released for soybean cyst nematode resistance whereas William 82 was released for Phytophthora root rot resistance. The information in Line 136 to 141 should be included on the top along with the parental line. It would be best for the authors to provide more background information for the readers to follow along.

Authors response:

We moved the information to the top along with the parental lines, as suggested. We included detailed about Forrest and Williams 82 in Materials and methods; we included a Figure (Fig 1) and supplementary information/file (S1_File) about the background of the parent and crosses for the benefit of the reader.

Reviewer’s comment

The cultivars in the study should include the PI number and provide citation
Line 127 The parental lines should include details of the parental lines such as the year that it was released, which research station developed the cultivar, agronomic traits such as flower color, growth habit, seed composition, maturity groups etc.

Authors response

We included a figure (Fig 1) and supplementary information/file (S1_File) about the background of the parent. We also detailed their background in the Materials and methods ( we included their PI, their crosses, Fig 1; S1_File) and more for the benefit of the reader. All are included in the text and all are track-changed.

Reviewer’s comment

Were the experiments conducted on university research farms? More details are needed.

Authors response

Thank you for the suggestion. Yes, the experiments were conducted on university research farms, as described in K (2021) [27]. We have updated the manuscript with more details and to clarify the experimental sites, including affiliations and research station details, to ensure full transparency and reproducibility. All new edits are track-changed.

Reviewer’s comment

Experimental design and replications are not discussed

Authors’ response

We thank the reviewer for pointing this out. The experimental design and replication details were originally described in Knizia et al. (2021), but we further included in the manuscript further details for clarity and completeness. We have revised the Materials and methods section to include a full description of the field design, replication, and environmental factors impacting data collection.

Reviewers’ response

Line 139 What type of amino acids?

Authors’ response

Amino acids included threonine, serine, proline, glycine, alanine, cysteine, valine, methionine, phenylalanine, lysine, tryptophan. We included them in the text, and all are track-changed.

Reviewer’s comments
The manuscript references many literatures but does not provide sufficient details for the readers to follow along. For example, it does not reference the BARCSoySNP6k Chip in the texts.
Inconsistency regarding the units throughout the article, some have space while others do not
Line 182 provide link to SoyBase

Authors’ response

-Thank you for the comment. We adjusted the text and provided further information and a citation and reference, below, for better clarity for the reader.

Soybean BARCSoySNP6K: An assay for soybean genetics and breeding research. The Plant Journal (2020) 104, 800–811 doi: 10.1111/tpj.14960.

-We provided the link

Reviewer’s comment

Statistical Analysis
Has the author compared different QTL mapping methods such as CIM vs Multiple QTL Mapping?

Authors’ response

No, we did not, and our reasons for this are below, and also some clarification was added to the Material and methods:

We appreciate the reviewer’s question regarding the QTL mapping approach. In this study, we used **Composite Interval Mapping (CIM)** implemented in **WinQTL Cartographer 2.5**, which is a widely accepted and robust method for detecting QTL with moderate to high power and resolution.

We acknowledge that alternative methods such as **Multiple QTL Mapping (MQM)** or **Inclusive Composite Interval Mapping (ICIM)** may offer complementary insights, particularly in separating closely linked QTL or modeling epistatic interactions. However, the current study focused exclusively on CIM, and we did not perform MQM analyses.

We have clarified this methodological choice in the revised **Materials and Methods** section, and we now explicitly note this as a limitation and a potential avenue for future research in the **Discussion** section. Our decision was based on the well-established performance of CIM for initial QTL discovery in biparental RIL populations and the consistency it offers across different traits and environments.

Reviewer’s comment

Tables lack units

Authors’ response

We revised the all tables and included the units as appropriate.

Reviewer’s comment

Please check if the SNP markers have a ss name in SoyBase.

Authors’ response

The SNP markers are in Soybase and have a ss name in SoyBase (Song et al., 2020. Plant J. 2020 Sep 23;104(3):800–811. doi: 10.1111/tpj.14960, and Akond et al., Please refer 2013. Journal of Plant Genome Sciences, Vol. 1 Not doi: 10.5147/jpgs.2013.0090. These references are included in the reference list.

Reviewer’s comment

The LOD score for Carbondale IL location is large, is there any reason behind this? Include linkage group information on the tables.

Authors’ response

We thank the reviewer for noting the unusually high LOD scores observed in the Carbondale, IL 2020 dataset. There might be several reasons for this, below, and we have now explained this in the text (Discussion):

1. **Larger Population Size (n = 306)**: Unlike the NC site in 2018, which had reduced data due to hurricane damage (n = 187), the IL 2020 trial included the full RIL population. The increased number of data points likely enhanced statistical power, reducing residual variance and inflating LOD values.
2. **Greater Trait Variation and Data Quality**: Environmental conditions in Carbondale were more stable, and no external damage affected plant performance. As a result, trait expression was clearer, and stronger marker-trait associations could be detected, especially for seed N, which showed high heritability (H² = 0.917).
3. **Narrow Confidence Intervals and High Additive Effects**: In several cases (e.g., qN-01-[IL-2020] and qN-03-[IL-2020]), the trait exhibited strong additive effects combined with tightly linked markers, further contributing to elevated LOD values.

We agree with the reviewer that including **linkage group (LG)** designations alongside chromosome numbers in the tables would enhance clarity. We have revised **Table 3** to include both chromosome numbers and corresponding LGs as per SoyBase nomenclature.

Reviewer’s comment

-The cultivar names in the manuscript should be consistent, for example Williams 82 only needs the ‘’ the first time it appears in the texts.

-Inconsistency in the way numbers are written and how the data is presented, Line 233 for example which chromosomes, is it seven chromosomes or Chr. 7?

-The range for the phenotypic variation in the Carbondale, IL location is rather large, is there a reason for that (7.14 – 78.52%)?
Line 259 qP-02 should be italicized
Line 349 F5:7

Authors’ response

We revised the manuscript, and addressed all comments above. We explained why the variation in Carbondale, IL, is rather large. We explained that the narrower or wider range of the phenotypic variation can be due to the environmental parameters of each year. The quantitative traits, such as nutrients, interact with the genotype differently. So, we expect a wider or narrower phenotypic variation that is caused by the uptake and assimilation of nutrients. We also commented on the environmental factors, including rainfall, wind, temperature caused by Hurricane Florence occurred in Spring Lake, NC, in 2018, and this could be a source of variance, in addition to the genotypic variance among the lines in each year

Reviewer’s comment

The discussion and candidate gene annotation section is well written. However, it does not conduct any studies to validate the QTL.

Authors’ response

We thank the reviewer’s for the comment, and it is valid. However, we did not conduct experiments to validate the QTL due to the following reasons:

-Limited resources (financially and human power).

-Validation of QTL needs a stand-alone project with separate project, and we are planning to conduct validation f QTL in the future projects.

- We viewed the attachment and addressed all comments in the attachment.

**We appreciate the reviewers’ comments, and we thank them for their time and efforts. We believe the reviewers’ comments improved the quality of our manuscript.**
